# SNAP25-induced MYC upregulation promotes high-grade neuroendocrine lung carcinoma progression

**DOI:** 10.3389/fimmu.2024.1411114

**Published:** 2024-10-04

**Authors:** Zhiqiang Chen, Shujing Wang, Jingrui Wang, Ying Wang, Xiangjun Qi, Bo An, Lingling Sun, Lizhu Lin

**Affiliations:** ^1^ The First Clinical Medical College, Guangzhou University of Chinese Medicine, Guangzhou, China; ^2^ Department of Oncology, The First Affiliated Hospital of Guangzhou University of Chinese Medicine, Guangzhou, China; ^3^ Guangdong Clinical Research Academy of Chinese Medicine, Guangzhou, China

**Keywords:** high-grade neuroendocrine carcinoma, synaptosome associated protein 25, c-Myc, MEK, ERK

## Abstract

**Background:**

This study investigated the expression and role of Synaptosome associated protein 25 (SNAP25) in high-grade neuroendocrine carcinoma (HGNEC).

**Methods:**

We used differentially expressed analysis and weighted gene co-expression network analysis (WGCNA) to identify key genes and modules in HGNEC. KEGG and GO analyses helped understand these genes’ roles, and ROC curves assessed their diagnostic value. We also studied SNAP25’s relation to immune infiltration and confirmed findings with *in vitro* and vivo experiments and datasets.

**Results:**

WGCNA identified 595 key genes related to pathways like MAPK signaling, GABAergic synapse, and cancer-related transcriptional misregulation. Top genes included SNAP25, MYC, NRXN1, GAD2, and SYT1. SNAP25 was notably associated with M2 macrophage infiltration. Dataset GSE40275 confirmed SNAP25’s high expression and poor prognosis in HGNEC. qRT-PCR and WB analyses showed increased SNAP25 and c-MYC levels in HGNEC, promoting MEK/ERK pathway activity. Reducing SNAP25 decreased H1299 cell proliferation, migration, invasion, and levels of c-MYC, MEK, and ERK. Finally, *in vivo* experiments further confirmed that SNAP25 knockout can inhibit tumor growth.

**Conclusion:**

SNAP25 regulates c-MYC activation by stimulating the MEK/ERK pathway, ultimately influencing the development of HGNEC.

## Introduction

Lung cancer stands as the foremost contributor to cancer-related fatalities on a global scale. Remarkably, approximately one-third of these tumors originate from neuroendocrine (NE) cells. Lung neuroendocrine tumors (NETs) are typically categorized using the World Health Organization’s three-grade(high, intermediate, low) classification system (1). Within this classification, high-grade neuroendocrine carcinoma (HGNEC) encompass small-cell lung carcinoma (SCLC) and large-cell neuroendocrine carcinoma (LCNEC), while intermediate- and low-grade lung NETs are further divided into atypical (AC) and typical (TC) carcinoids (2). Despite their histopathological distinctions, both types of HGNEC exhibit a grim clinical trajectory, marked by a 5-year survival rate ranging from 15 to 57% (3–5). Notably, HGNEC exhibit a more rapid growth rate than other forms of lung cancer, manifesting as particularly aggressive and carrying a comparatively poor prognosis. In the current study, the scarcity of investigations examining the prognosis of HGNEC underscores the critical need to delve deeper into the molecular mechanisms governing their development and progression. This pursuit can potentially lead to the discovery of novel treatment targets and strategies aimed at enhancing the overall prognosis of afflicted patients.

Synaptosome associated protein 25(SNAP25) is a soluble component of the N-ethylmaleimide-sensitive factor attachment protein receptor complex, playing an indispensable role in the regulation of various crucial cellular processes. These include neurotransmitter release, synaptic messaging, secretory vesicle extravasation, intercellular signaling, and ion channel opening (6–8). SNAP25 has also been implicated in facilitating normal vesicle fusion and orchestrating lysosomal trafficking (9, 10). In recent years, a growing body of research have underscored an association between SNAP25 levels and various types of tumors (11–15). Nevertheless, the precise functions and mechanisms underlying SNAP25’s involvement in the immunologic and pathogenetic progression of high-grade neuroendocrine lung cancer remain largely unexplored.

MYC, a pivotal regulatory gene in tumorigenesis, exhibits significantly elevated expression levels in various tumor types. Its gene product exerts control over the transcription of an array of protein-encoding genes and non-coding RNAs (16). Meanwhile, the ERK/MAPK represents a crucial signaling cascade with a pivotal role in governing numerous cellular processes, encompassing cell proliferation and apoptosis (17, 18). Prior investigations have highlighted that MYC’s capacity to activate the ERK/MAPK signaling pathway, thereby promoting the proliferation of NSCLC cells while inhibiting apoptosis. However, there is a noticeable absence of comprehensive studies delving into the distinct characteristics of HGNEC in this context (19).

In pursuit of this objective, we accessed gene expression data from the GEO database and conducted survival analyses to assess the prognostic significance of gene expression across diverse HGNEC cohorts. Notably, our findings highlighted the increasingly prominent role of SNAP25 protein became more pronounced as the prognosis of patients with HGNEC patients worsened, the SNAP25 as patient prognosis deteriorated, solidifying its status as a valuable prognostic biomarker. This designation prompted us to embark on a comprehensive analysis. Our approach encompassed integrative analysis and the utilization of various visualization techniques to delve into the mechanisms governed by SNAP25 in HGNEC. We found a close association between SNAP25 and its downstream protein, c-MYC. We meticulously examined the correlation between the expression levels of SNAP25 and c-MYC and established a correlation between SNAP25 expression and the immune microenvironment of patients. Furthermore, we harnessed gene ontology (GO) analysis, explored Kyoto Encyclopedia of Genes and Genomes (KEGG) and employed various methodologies to unravel the potential mechanisms underpinning SNAP25’s involvement in tumor development. Subsequently, we conducted *in vitro* and vivo experiments to validate and further illuminate the role of SNAP25-induced upregulation of c-MYC in the progression of HGNEC, offering novel insights into its pivotal significance within this context.

## Materials and methods

### Data source and differentially expressed genes identification

This dataset was generated by profiling the transcriptomes of high-grade neuroendocrine carcinoma (HGNEC) from a cohort of 29 patients who underwent pneumoresection. Within this patient group, 20 individuals were diagnosed with small cell lung carcinoma (SCLC) while the remaining 9 had large cell neuroendocrine carcinoma (LCNEC). A classification process, guided by transcription profiles and statistical analysis, segregated the samples into two distinct groups, denoted as group 1 and group 2. Notably, the analysis revealed that group 2 exhibited a notably higher survival rate compared to group 1. The expression data is available for download via the Gene Expression Omnibus (GEO) database at the following link: https://www.ncbi.nlm.nih.gov/geo/query/acc.cgi?acc=GSE9074.

To initiate our analysis, we utilized R software, specifically version 3.6.1, to import and preprocess the dataset GSE9074. This preprocessing involved essential steps such as batch correction and normalization. Subsequently, we conducted DEG analysis screening, employing the “limma” package for this purpose. DEGs that met the criteria of |log2FC|>2 and an adjusted p-value<0.01 were carefully selected for further investigation. Following the significance analysis of expression levels, we harnessed the “pheatmap” and “ggplot2” R packages (20) to create visually informative volcano plots and DEG expression heat maps.

### Candidate hub genes screen and functional enrichment analysis

WGCNA, as a systematic approach in the field of biology, is frequently employed to elucidate patterns of genetic association among diverse samples (21). It serves as a valuable tool for identifying highly co-regulated genomes. By analyzing the interconnections between genomes and their relationships with phenotypic traits, this approach enhances the identification of candidate markers. In our study, we leveraged the “WGCNA” R package to construct a gene co-expression network specifically tailored to HGNEC. Subsequently, we evaluated the correlation of different modules within this network with the pathogenic mechanism associated with HGNEC. We identified the most relevant module, which yielded the central gene derived from our WGCNA analysis.

To identify key genes relevant to the pathogenesis of HGNEC, we selected the genes that intersected between WGCNA’s most relevant module genes, and the genes identified as differentially expressed (DEGs). These intersecting genes were then designated as candidate hub genes pertinent to HGNEC pathogenesis. Subsequently, we performed Gene Ontology (GO) and Kyoto Encyclopedia of Genes and Genomes (KEGG) enrichment analysis, employing the “clusterProfiler” R package. This analysis aimed to provide insights into the potential mechanisms governing the progression and pathogenesis of HGNEC. Additionally, we utilized the KEGG database to elucidate the biological functions associated with the significant hub genes.

### Protein-protein interaction network hub genes and receiver operator characteristic curve

After disregarding the disconnected nodes and screening for relevant DEGs using an interaction score >0.4, hub genes were screened using STRING (https://string-db.org/) and Cytoscape software platform (https://cytoscape.org/) to show the protein-protein interaction (PPI) networks. The Degree algorithm of Cytoscape software was used to rank the important genes in PPI networks using the cytoHubba plugin. After that, we used “ROC” package construct the receiver operator characteristic (ROC) curve to validate the diagnostic effectiveness of the candidate biomarkers. We employed the area under the ROC curve (AUC) to indicate the accuracy. A criterion (0.9≤AUC<1) was used to identify excellent accuracy.

### Analysis of immune cell infiltration and its relationship with hub genes

To explore the role of immune cells in HGNEC, we conducted an analysis to assess the extent of immune cell infiltration among 22 distinct types of immunocytes in the two groups using CIBERSORT. Additionally, we examined the correlation between these hub genes and immune infiltrates, encompassing neutrophils, CD8^+^ T cells, CD4^+^ T cells, dendritic cells, B cells, and macrophages in HGNEC.

### Validation analysis using GSE40275 dataset

To validate our findings, we performed an additional analysis using an independent dataset, GSE40275. This dataset includes RNA samples extracted from lung tissues, comprising 15 cases of small cell lung cancer (SCLC) and 43 samples from individuals with healthy, normal lung tissue. The GSE40275 dataset was downloaded from the Gene Expression Omnibus (GEO) database. We conducted differential expression analysis to identify genes with significant expression changes between SCLC and normal lung tissues. To further explore the clinical significance of SNAP25, we performed a survival analysis focusing on its expression levels in high-grade neuroendocrine carcinoma (HGNEC) of the lung. Kaplan-Meier survival curves were generated, and the log-rank test was used to assess the statistical significance of survival differences between groups.

### Cell line culture and establishment of stable cell lines

H1299 (LCNEC), A549 (Lung adenocarcinoma cell), H520(Lung squamous cell) and BEAS-2B (Human normal lung epithelial cell line) cells were purchased from American Type Culture Collection (ATCC, USA). Cell lines have recently been identified using the STR method. The H1299 and A549 cells were cultured in RPMI-1640 medium (Sigma, Saint Louis, MO, USA) with 10% fetal bovine serum (FBS; Gibco, Rockville, MD, USA), 100 U/mL penicillin and 100 μg/mL streptomycin (Gibco, Rockville, MD, USA) at 37°C in an environment with 5% CO2. H520 and BEAS-2B cells were maintained in Dulbecco’s modified Eagle’s medium (DMEM; Gibco, Rockville, MD, USA) containing 10% FBS, 100 U/mL penicillin and 100 μg/mL streptomycin at 37°C in an environment with 5% CO2.

Lentiviral constructs of SNAP25-related knockdown plasmids and their corresponding empty vectors were purchased from Shanghai GeneChem Co.,Ltd. All the plasmids were amplified and extracted using HGNEC cell lines seeded in 6-well plates at a concentration of 2×10^6^ cells per well before lentivirus infection. When the confluence reached approximately 50%, the H1299 cell lines were infected with the lentiviruses. After 48 hour of infection, the cells were selected with puromycin (Beyotime Biotechnology, Shanghai, China).

### Cell counting kit-8 assay

A total of 5×10^3^ H1299 cells infected with the lentiviruses were first seeded into each well of a 96-well plate and subjected to the specified experimental treatment. The next day, the cell medium was changed, and the cells were further cultured for 24, 48, and 72 hours respectively. After this period, the original medium was removed. Subsequently, 100 μL of fresh medium and 10 μL of CCK-8 reagent (Dojindo Molecular Technologies, Kumamoto, Japan) were added to each cell-containing well, followed by incubation at 37°C for an additional 2 hours. Post-incubation, the optical density of each sample in the 96-well plate was measured using a multifunctional microplate reader (Bio-Rad, Hercules, CA, USA) at a wavelength of 450 nm.

### Migration and invasion assay

Initially, H1299 cells infected with the lentiviruses underwent trypsinization and were suspended in a serum-free medium. Subsequently, a 100 μL sample of this cell suspension (containing 1 × 10^5^ cells) was placed into the Transwell’s upper compartment (Corning Costar, MA, USA), which features an 8 μm pore-sized porous polycarbonate membrane. The lower compartment was filled with 600 μL of a medium enriched with 10% FBS. Following a 24-hour incubation, cells remaining on the membrane filter’s top surface were carefully removed using cotton swabs. Cells that had migrated to the underside of the filter were treated with 4% methanol, then stained with a 0.5% crystal violet solution for two hours, rinsed in PBS, quantified, and examined under a microscope. In the invasion assay, the Transwell’s upper section was lined with 60 µL of Matrigel (1:20 dilution, Corning Costar, MA, USA). Cells that penetrated to the filter’s lower surface were processed and analyzed as previously mentioned.

### Nude mouse tumor xenograft model

Animal experiments were approved by Guangzhou University of Chinese medicine animal center. The BALB/C nude mice (6-week-old) were obtained from Guangdong Yaokang Biotechnology Co., LTD. and were randomly divided into two groups (n = 6 per group) and stably transduced H1299 cells (1 ×10^7^) were subcutaneously injected into the unilateral hind limbs of the nude mice. Tumor size was measured for 21 days after inoculation to calculating tumor volume using the equation (length × width^2^/2). Animals were killed 21 days after inoculation, and the tumors were then excised and weighted.

### qRT-PCR assay

Total RNA was extracted from the tissue and cells using the Trizol Reagent (Thermo Fisher Scientific, Waltham, MA, USA) in accordance with the manufacturer’s protocol. Subsequently, the reverse transcription was carried out utilizing Maxima Reverse Transcriptase (Thermo Fisher Scientific, Waltham, MA, USA). Quantitative PCR was executed using SYBR premix Ex Taq (TaKaRa Biotechnology Co., Ltd., Dalian, China) and performed on QuantStudio™ 5 Real-Time PCR System (Thermo Fisher Scientific, Waltham, MA, USA). Relative expression levels were normalized to GADPH and calculated using the 2^-ΔΔCt^ method. Each experiment was conducted independently with a minimum of three replicates. The primers are as follows: GAPDH-F, TGTCAAGCTCATTTCCTGGTATG, R TCTCTCTTCCTCTTGTGCTCTTG. SNAP25-F GGTAACAAATGATGCCCGAGAAA, R ACTTAACCACTTCCCAGCATCTT. MYC-F CAAGAGGCGAACACACAACG, R GTCGTTTCCGCAACAAGTCC. ERK1/2-F GTGTTGCAGATCCAGACCATGAT, R TGCAGCCTACAGACCAAATATCA. MEK-F CCCTCCAACATCCTAGTCAACTC, R ATCTGGAGGAGGGATGGGATAC.

### Western blot analysis

Total protein was extracted from the tissue and cells using RIPA lysis buffer (Beyotime, Shanghai, China) according to the manufacturer’ s guidelines. Then, protein concentration was measured by the bicinchoninic acid (BCA) protein assay kit (Beyotime Biotechnology, Shanghai, China) according to the instructions of the kit. Subsequently, the proteins were separated using 10% sodium dodecyl sulphate (SDS)-polyacrylamide gel electrophoresis (PAGE) and then transferred to polyvinylidene fluoride membrane (PVDF; Roche, Basel, Switzerland). After blocking with QuickBlock Blocking Buffer (Beyotime, Shanghai, China) at room temperature for 15min, the membranes were incubated with the relevant antibodies overnight at 4°C. After a thorough wash with 1 × PBS, the membranes were probed with HRP-conjugated secondary antibodies for 1 hour at room temperature, Primary antibodies for SNAP25 (1:3000), p-MEK (1:3000), MEK (1:3000), p-ERK (1:3000) and ERK (1:3000) were purchased from Affinity. Primary antibodies for c-MYC (1:5000) and GAPDH (1:3000) were obtained from proteintech. Specific protein bands were detected utilizing an ECL western blotting substrate (Bio-Rad, Hercules, CA, USA) and the Fluorescence/Chemiluminescence Imaging System (CLINX Science instruments, Shanghai, China), with subsequent analysis using Image J software. Each experiment was repeated at least three times.

### Immunofluorescence

Immunofluorescence staining was performed to assess the expression of SNAP25 in H1299, A549, H520 and BEAS-2B cells. In brief, cells were fixed using 4% paraformaldehyde for 15 min, permeabilized with 0.5% Triton X-100 for 15 min, and subsequently blocked with goat serum. Following this, cells were subjected to overnight incubation at 4°C with an anti-SNAP25 (1:300, Cell Signaling Technology) antibody, followed by incubation with an FITC-conjugated secondary antibody (ZSGB-BIO) in the dark for 1 hour. Cell imaging was performed utilizing an immunofluorescence inverted microscope (Leica Dmi8, Wetzlar, Germany). The expression of SNAP25 was quantified using the average fluorescence intensity values determined by the Image J software.

### Statistical analysis

Data were analyzed with the Statistical Package for the Social Sciences (SPSS 26.0) and were performed primarily using Image J software. Statistical significance was determined with a threshold of P-value<0.05. Visualization of results was achieved using GraphPad Prism 9.2.0 (La Jolla, CA, USA). To ensure the robustness of our findings, all experiments were independently repeated three times for validation.

## Results

### DEGs screening

**Figure 1:**
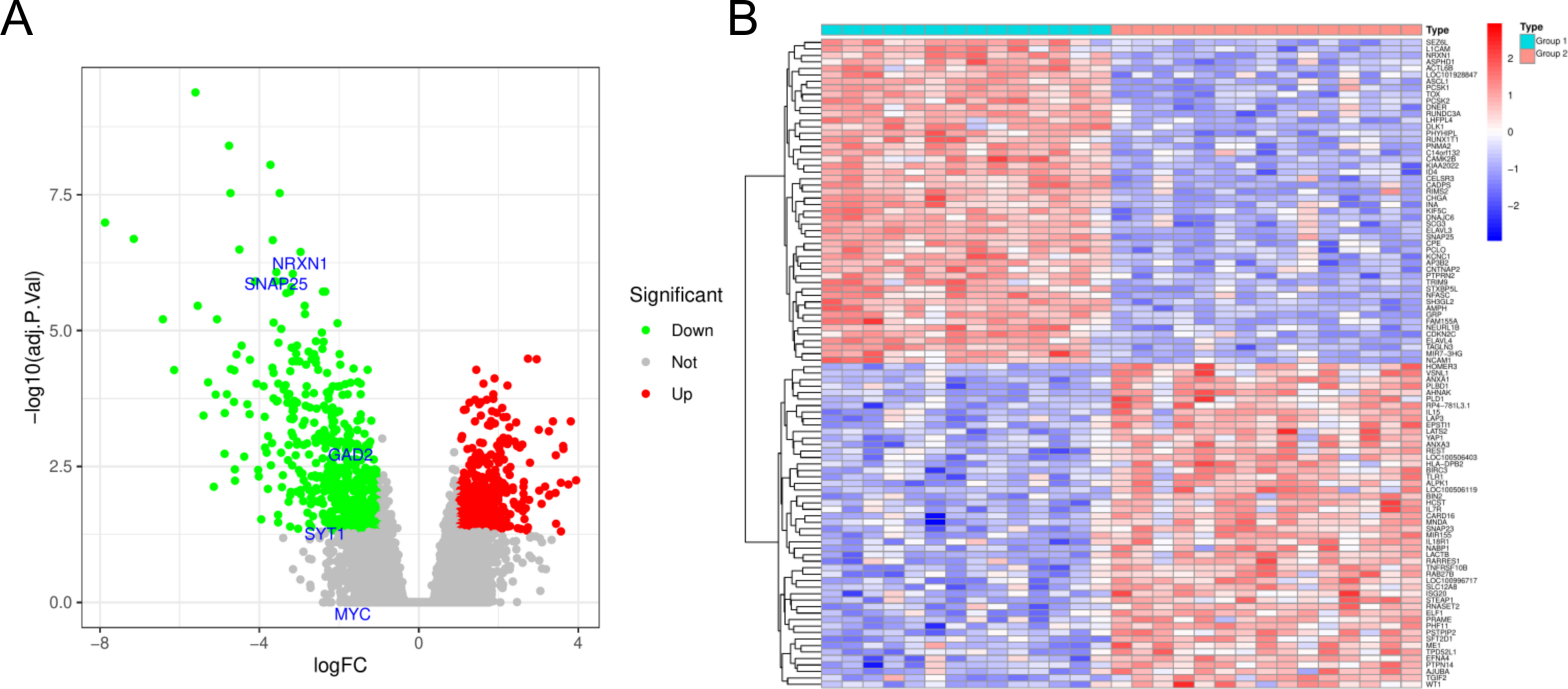
Genes differentially expressed between the Group 1 and Group 2. **(A)** Volcanic map for differential expression analysis of GSE9074. **(B)** Heat map for differential expression analysis of GSE9074. Blue represents down-regulated genes and red represents up-regulated genes.

Our initial step involved acquiring the HGNEC dataset (GSE9074) from GEO database. Within this dataset, patients were categorized into two distinct groups based on their transcriptomic profiles. Group 1 consisted of patients with a lower survival rate, while Group 2 included those with a significantly higher survival rate. We proceeded to identify DEGs between these two groups. Our analysis revealed a total of 941 DEGs, with 426 genes being upregulated and 515 downregulated in Group 2 compared to Group 1 (**Figures 1A, B**; **Supplementary Table S1**).

### WGCNA network construction and identification of modules related to HGNEC

**Figure 2:**
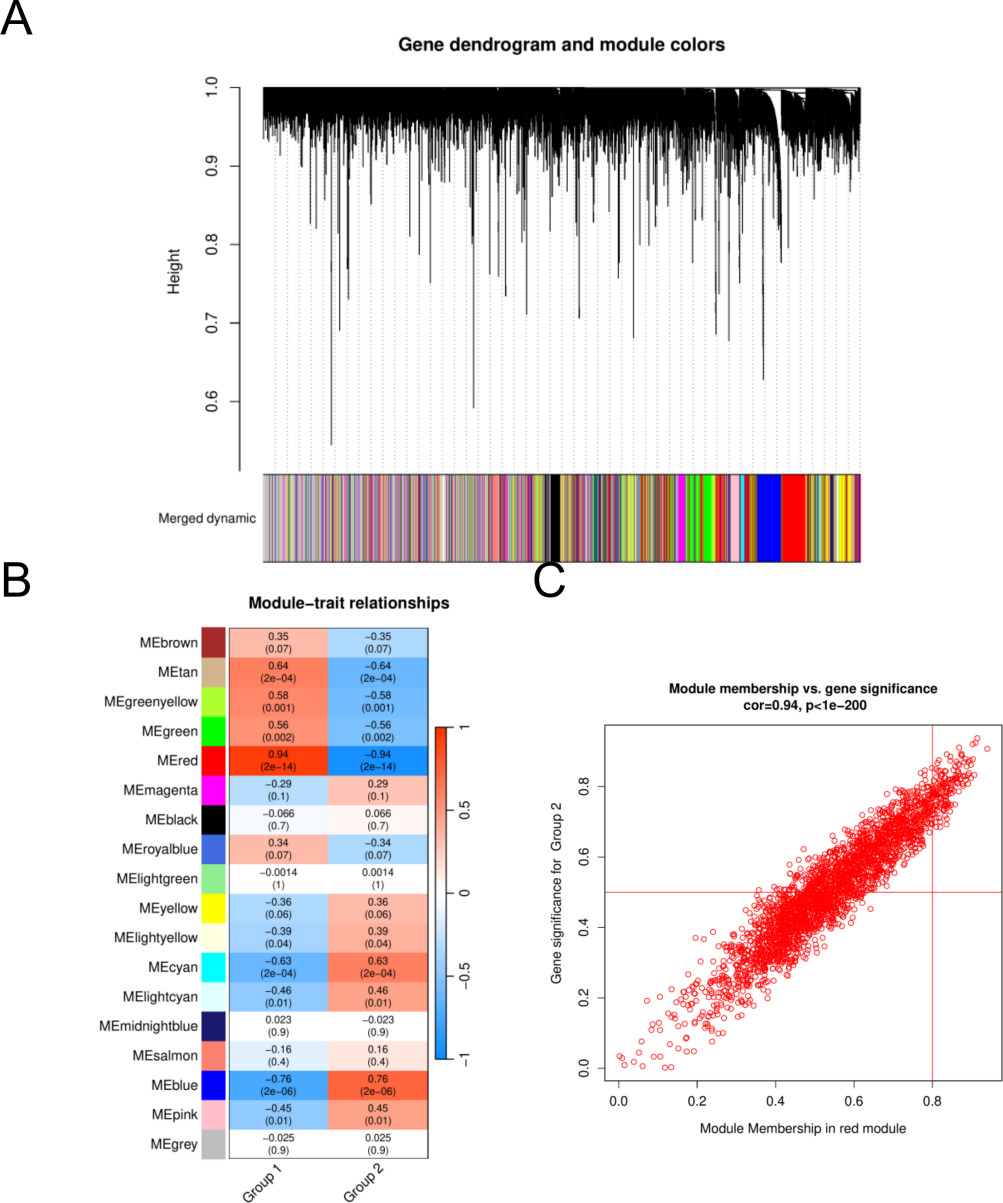
Identification of HGNEC -associated gene modules in the GEO dataset using WGCNA. **(A)** Dendrogram of all genes in the GSE9074 dataset was clustered on the basis of a topological overlap matrix. Each branch in the clustering tree represents a gene, while co-expression modules were constructed in different colors. **(B)** Module-trait heatmap of the correlation between the clustering gene module and neuropathic pain in the GSE9074 dataset. Each module contains the corresponding correlation coefficient and p value. **(C)** Scatter plot of module cyan has the strongest positive correlation with HGNEC in the GSE9074 dataset.

To assess the potential associations of gene modules with HGNEC, we conducted WGCNA analysis utilizing all candidate genes from HGNEC -related datasets, specifically GSE9074 (**Figure 2A**). In the course of this analysis, we identified a total of eighteen distinct modules (**Figure 2B**). Following a thorough examination of the positive correlation coefficients, we ultimately singled out module red as the most pertinent module within the GSE9074 dataset. (**Figure 2C**, **Supplementary Table S2**).

### Go/KEGG analyses

**Figure 3:**
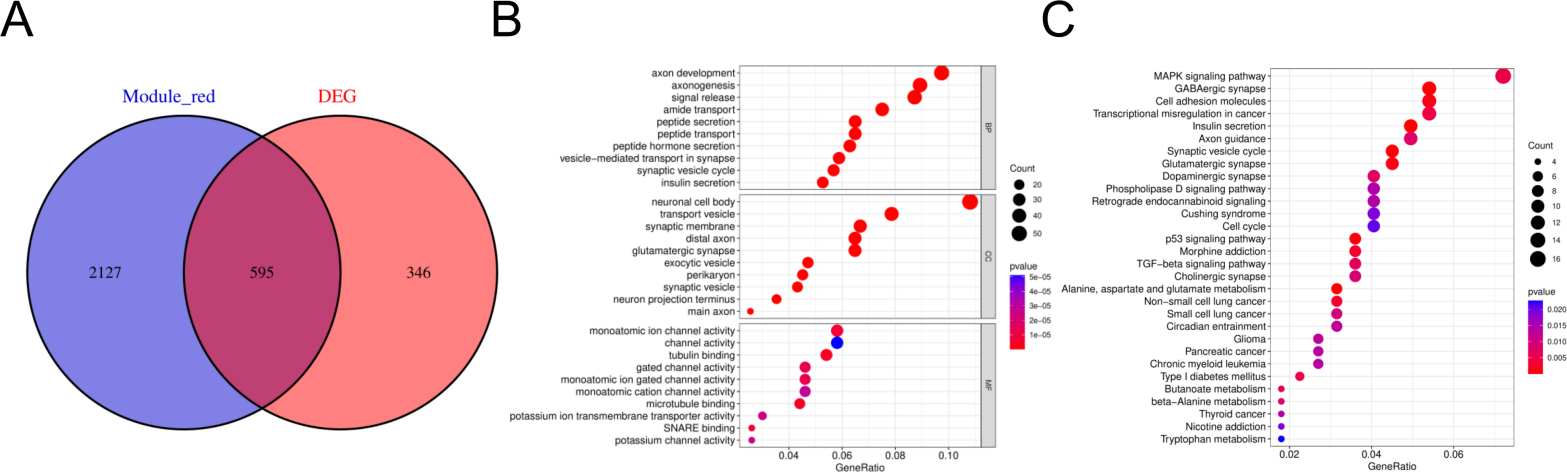
Candidate hub genes were screened and validated. **(A)** Venn diagram revealed 595 overlapping candidate hub genes. **(B)** GO enrichment analysis of candidate hub genes. **(C)** KEGG pathway analysis of candidate hub genes.

To search for co-expressed genes between WGCNA-derived hub genes and DEGs. We eventually screened 595 overlapping genes as candidate hub genes that may play an important role in the development and progression of HGNEC (**Figure 3A**). GO and KEGG analyses were conducted to further explore the underlying roles of these 595 overlapping genes (**Figures 3B, C**). GO enrichment analysis showed that the overlapping genes mainly affect the biological functions of axon development, axonogenesis, neuronal cell body and monoatomic ion channel activity. KEGG enrichment analysis showed that the overlapping genes mainly affect MAPK signaling pathway, GABAergic synapse, cell adhesion molecules and transcriptional misregulation in cancer.

### PPI network analysis and ROC curves for hub genes

**Figure 4:**
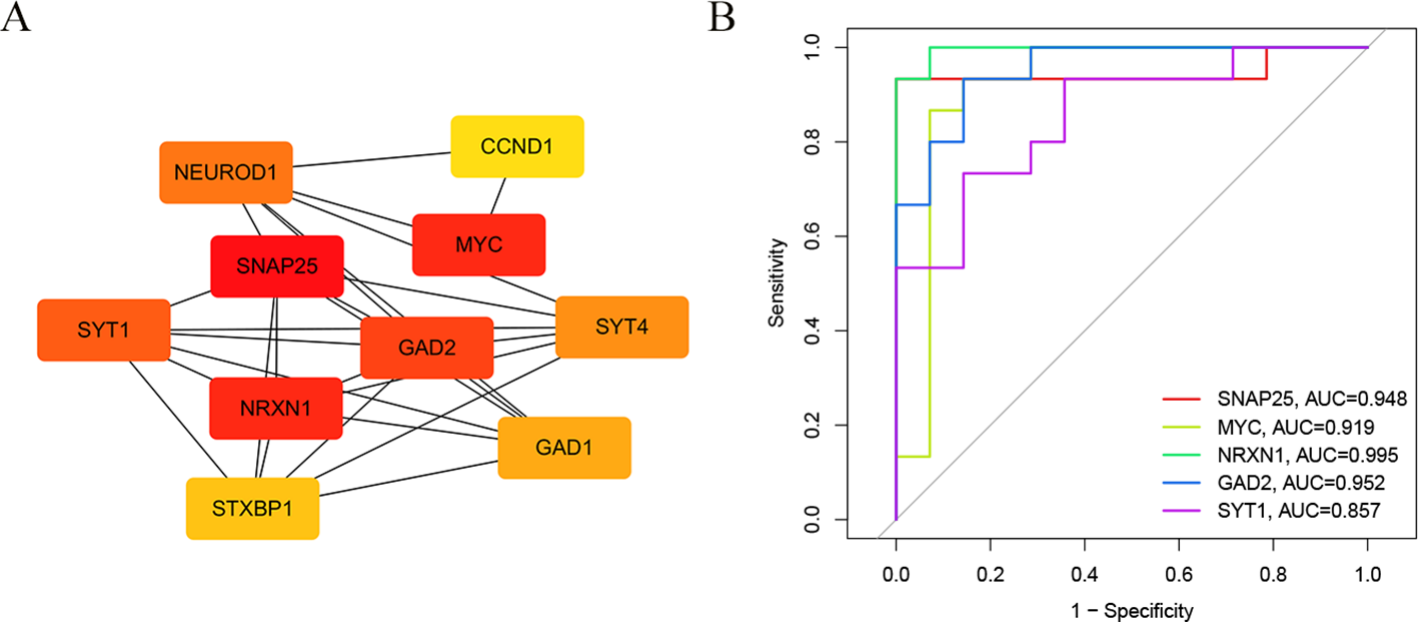
The construction of PPI network and ROC curves. **(A)** The core genes of the interaction network were obtained by degree algorithm. **(B)** ROC curves to assess the diagnostic efficacy of each hub gene.

We used the STRING online tool to construct a PPI network of overlapping hub genes. Subsequently, the top ten highly ranked up-regulated genes were then visualized by using Cytoscape software (**Figure 4A**). Briefly, SNAP25, MYC, NRXN1, GAD2, SYT1, GAD1, SYT4, NEUROD1, CCND1, and STXBP1 were sorted out. The deeper the color, the higher the score. Subsequently, we calculated ROC curves for the top five hub genes (SNAP25, MYC, NRXN1, GAD2 and SYT1) to assess the diagnostic effect. The AUC of our ROC curves could differentiate between group 1 and group 2 (**Figure 4B**). The AUC values of SNAP25, MYC, NRXN1, GAD2 and SYT1 are, respectively, 0.948, 0.919, 0.995, 0.952 and 0.857.

### Correlation between SNAP25 and immune cells and data set validation

**Figure 5:**
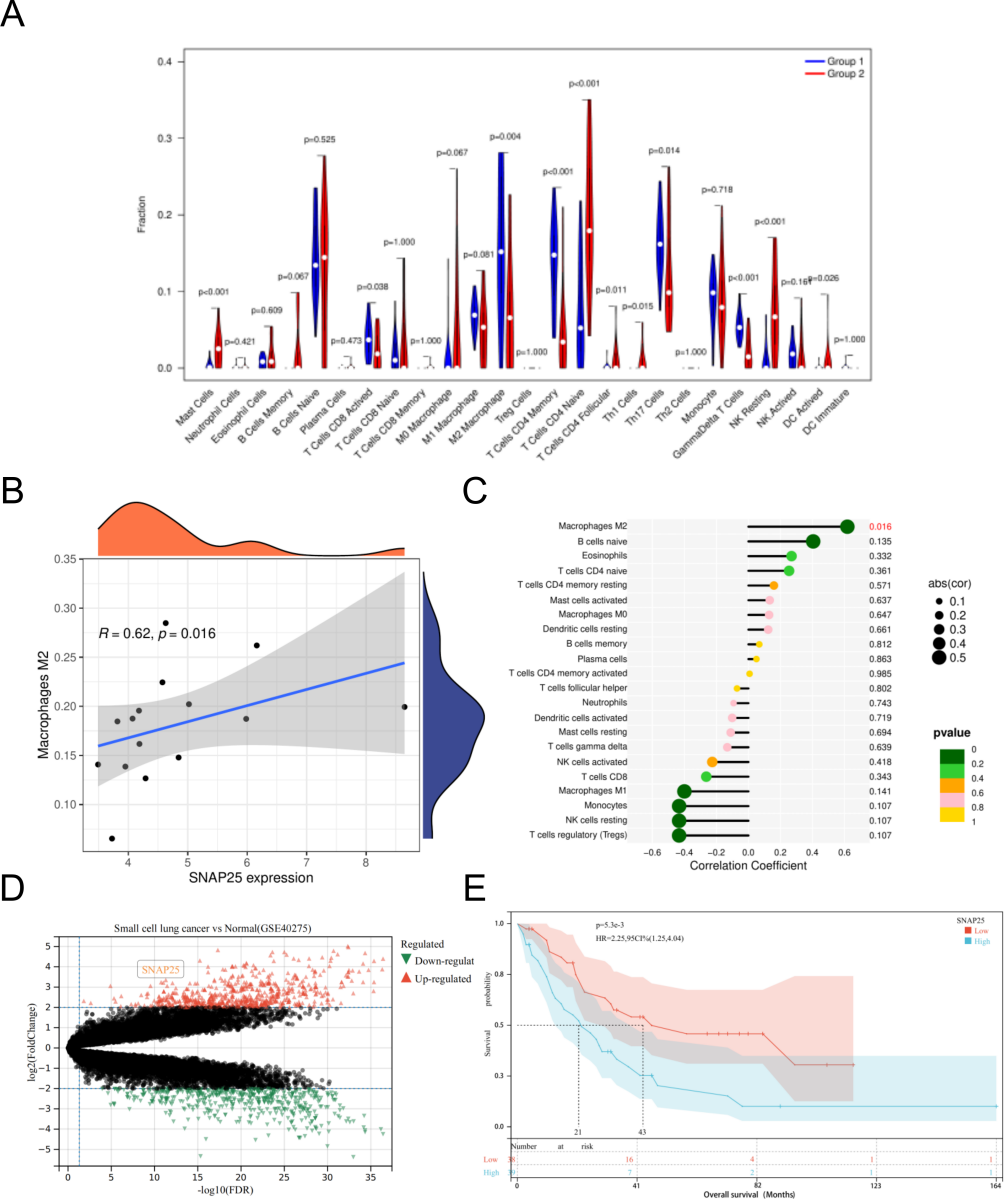
Immuno-correlation of SNAP25 in HGNEC. **(A)** Relative distribution of 22 kinds of immune cells in HGNEC samples. **(B)** The correlation between SNAP25 expression and M2 macrophages. **(C)** The correlation between SNAP25 expression and various immune cells. **(D)** DEGs in small cell lung cancer and normal lung RNA samples were analyzed based on data set GSE40275. **(E)** Prognostic analysis of SNAP25 in HGNEC.

Among the 22 immune cells types we analyzed, a substantial correlation emerged between the infiltration levels of M2 macrophages and SNAP25 expression. This correlation displayed a consistent upward trend with increasing SNAP25 expression levels. Notably, it’s worth highlighting that no significant correlations were detected between SNAP25 and other immune cell types. (**Figures 5A–C**).

Simultaneously, we utilized an additional dataset (GSE40275) to perform further validation analysis. This dataset consisted of RNA samples extracted from lung tissues, comprising 15 cases of small cell lung cancer and 43 individuals with healthy, normal lung tissue. In this analysis, we observed a significant upregulation of SNAP25 in small cell lung cancer compared to normal lung tissue (**Figure 5D**). Furthermore, we conducted a survival prognosis analysis specifically focusing on SNAP25 in HGNEC. The results indicated that individuals with elevated expression levels of SNAP25 experienced shorter survival periods (**Figure 5E**).

### Significant upregulation of SNAP25 gene expression at the cellular level in H1299 cells

**Figure 6:**
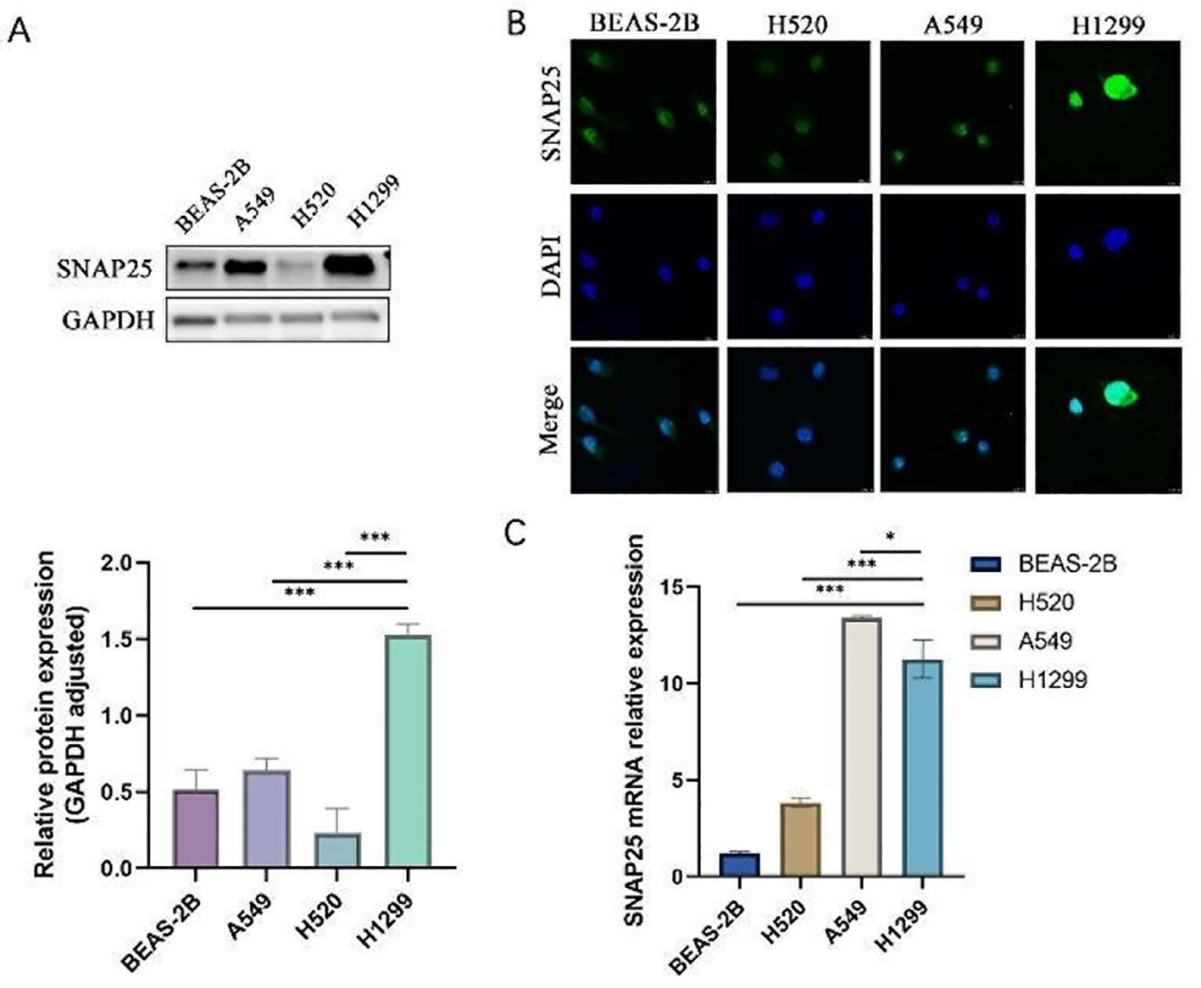
The expression of SNAP25 protein and mRNA among various cells. **(A)**SNAP25 protein expression among different cells. **(B)** Immunofluorescence analysis of SNAP25 expression among different cells. **(C)**SNAP25 mRNA expression among different cells.

To further investigate the role of SNAP25 in HGNECs, we selected various lung cancer cell lines for evaluation. These included the large cell lung cancer cell line H1299, the lung adenocarcinoma cell line A549, the lung squamous cell carcinoma cell line H520, and the normal lung epithelial cell line BEAS-2B. The qRT-PCR findings revealed that SNAP25 mRNA expression in H1299 cells was higher compared to BEAS-2B and H520 cells, but lower than that in A549 cells, and these differences were statistically significant (**Figure 6C**). These results were consistent with the observations from the GEO database, demonstrating that SNAP25 expression at the protein level was significantly elevated in neuroendocrine cancer cells with a high malignancy profile compared to lung cancer cells with relatively lower malignancy (**Figure 6A**) (1.529 ± 0.041 vs 0.643 ± 0.044 vs 0.234 ± 0.091 vs 0.518 ± 0.074, H1299 vs A549 vs H520 vs BEAS-2B).

To complement our findings, we conducted immunofluorescence experiments, which further substantiated a significant increase in SNAP25 expression levels specifically in H1299 cells (**Figure 6B**). Taken together, these results strongly indicate that elevated SNAP25 expression is indicative of a poor prognosis for HGNECs.

### Prominent activation of MEK/ERK/c-MYC pathway in H1299 cells at cellular level

**Figure 7:**
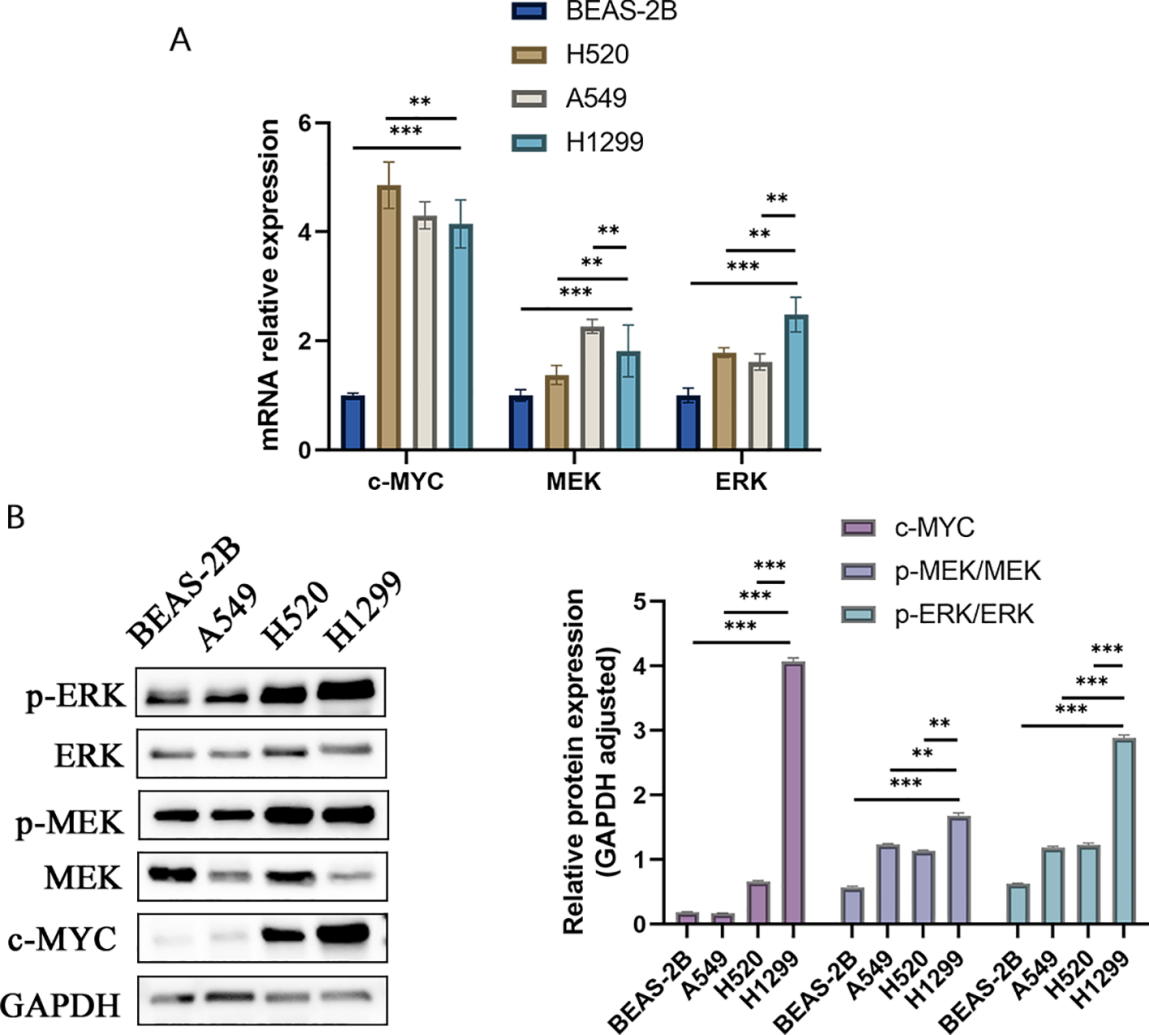
The expression of MEK/ERK pathway protein and mRNA among various cells. **(A)** MEK/ERK pathway mRNA expression among different cells. **(B)** MEK/ERK pathway protein expression among different cells.

The KEGG pathway analysis underscored the predominant association of these hub genes with the MAPK pathways. A closer look at the qRT-PCR data revealed that c-MYC mRNA levels in H1299 cells were higher than in BEAS-2B cells, but slightly lower compared to H520 and A549 cells. At the protein level, the expression of c-MYC in H1299 cells was significantly higher than that in other cell lines (4.068 ± 0.061 vs 0.171 ± 0.002 vs 0.660 ± 0.015 vs 0.191 ± 0.004, H1299 vs A549 vs H520 vs BEAS-2B). MEK mRNA expression in H1299 cells exceeded that in BEAS-2B and H520 cells and was marginally less than in A549 cells. Notably, the expression of p-MEK/MEK proteins in H1299 cells surpassed that in all other examined groups (1.683 ± 0.039 vs 1.237 ± 0.009 vs 1.138 ± 0.010 vs 0.572 ± 0.018, H1299 vs A549 vs H520 vs BEAS-2B). When it comes to ERK expression, H1299 cells exhibited higher levels than the other groups, both at the mRNA and protein levels (2.886 ± 0.045 vs 1.187 ± 0.019 vs 1.226 ± 0.029 vs 0.625 ± 0.010, H1299 vs A549 vs H520 vs BEAS-2B). To summarize, the MEK/ERK pathway activation appeared significantly more elevated in lung cancer cells with higher malignancy (**Figures 7A, B**).

### SNAP25 knockdown inhibits cell proliferation, migration, and invasion while modulating the MEK/ERK signaling pathway

**Figure 8:**
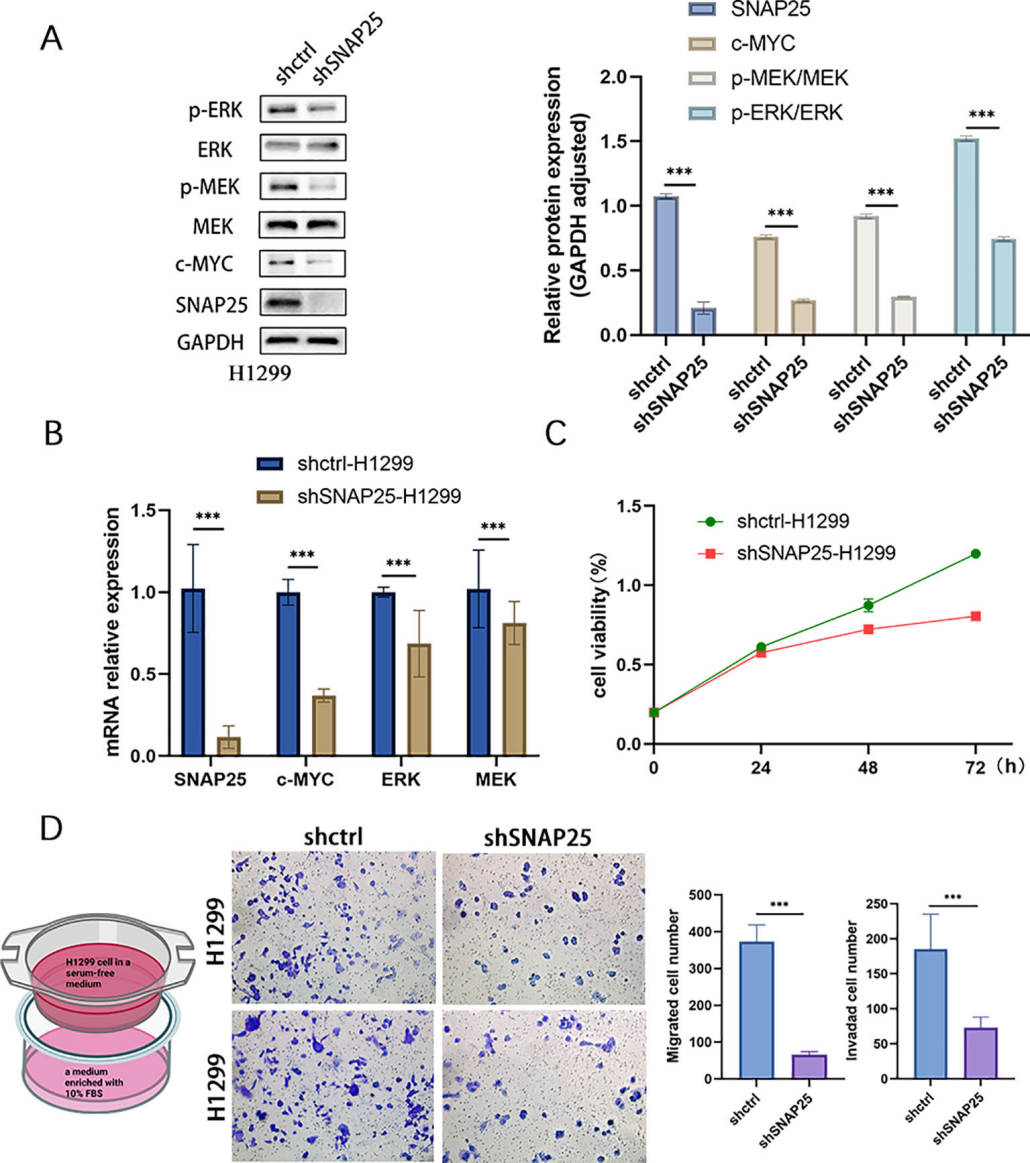
Comprehensive Analysis of SNAP25 knockout cells: impact on proliferation, mRNA expression, migration, invasion, and MEK/ERK pathway activation. **(A)** MEK/ERK pathway protein expression among control and SNAP25 knockout cells. **(B)** Changes in mRNA levels of SNAP25 knockout cells. **(C)** CCK8 results showed the proliferation of SNAP25 knockout cells. **(B)** Changes in mRNA levels of SNAP25 knockout cells. **(D)** Cell migration and invasion of SNAP25 knockout cells.

To further substantiate the correlation between SNAP25 and the MAPK signaling pathway, we initiated experiments by suppressing SNAP25 expression in H1299 cells using lentivirus-mediated transfection. PCR analysis further indicated that, in the SNAP25 knockdown group, key pathway components including c-MYC, MEK and ERK underwent notable modulation (**Figure 8B**). These alterations were corroborated at the protein level as well (**Figure 8A**) (SNAP25: 1.076 ± 0.019 vs 0.209 ± 0.047, shctrl vs shSNAP25) (c-MYC: 0.763 ± 0.014 vs 0.269 ± 0.011, shctrl vs shSNAP25) (p-MEK/MEK: 0.920 ± 0.018 vs 0.298 ± 0.005, shctrl vs shSNAP25) (p-ERK/ERK: 1.522 ± 0.019 vs 0.746 ± 0.015, shctrl vs shSNAP25). The CCK8 assay revealed that SNAP25 knockdown markedly reduced cell proliferation compared to the control group with empty vector transfection (**Figure 8C**). Additionally, Transwell assays demonstrated that cells with SNAP25 knockdown exhibited significant reductions in migration and invasion (**Figure 8D**).

### SNAP25 downregulation suppresses *in vivo* HGNEC xenograft growth

We further evaluated the impact of SNAP25 on the growth of HGNEC xenografts *in vivo*. For this purpose, stably transduced H1299 cells (1×10^7^ cells per condition) were subcutaneously injected into the flanks of nude mice to establish the xenograft models. Tumor growth was meticulously monitored every three days using calipers to measure the dimensions of the tumors. After three weeks, the mice were sacrificed, and the tumors were dissected and weighed.

**Figure 9:**
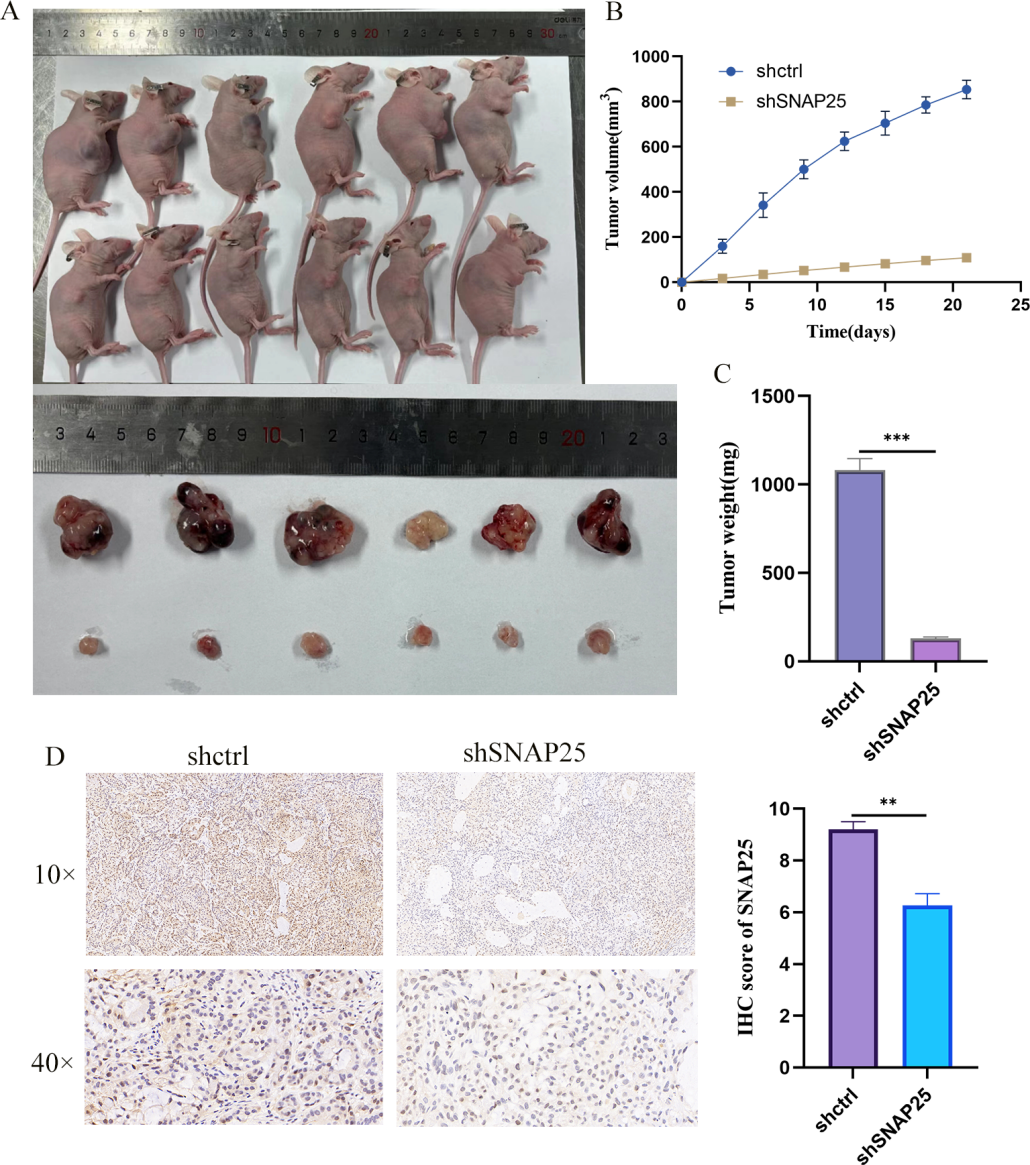
*In vivo* experiments further verified the differences of SNAP25. **(A)** Tumor growth of mice with subcutaneous tumor transplantation *in vivo*; **(B)** Tumor body differences between the two groups of mice; **(C)** The difference in tumor weight between the two groups. **(D)** Immunohistochemical analysis of SNAP25 expression *in vivo* models.

As depicted in **Figure 9A**, the downregulation of SNAP25 significantly inhibited tumor growth compared to the negative control group. The tumors in the SNAP25 downregulated group were visibly smaller and fewer in number. Tumor volume measurements, shown in **Figures 9B, C**, confirmed that SNAP25 downregulation effectively suppressed tumor growth post-xenografting, with a marked reduction in tumor volume and weight. Immunohistochemistry also further confirmed the expression of SNAP25 among the groups (**Figure 9D**) (SNAP25: 9.200 ± 0.173 vs 6.267 ± 0.260, shctrl vs shSNAP25). This data strongly suggests that SNAP25 plays a critical role in promoting the growth of HGNEC tumors *in vivo*.

## Discussion

HGNEC of the lung, such as SCLC and LCNEC, are rare, highly aggressive lung cancers originating from neuroendocrine cells (22). These cancers exhibit distinct biological and molecular characteristics due to their neuroendocrine origin, including marked invasiveness. HGNECs are identified by specific neuroendocrine markers like chromogranin A(CgA), neuron-specific enolase (NSE), synaptophysin, and CD56 (23, 24). They also show unique genetic mutations; SCLC frequently has TP53 and RB1 mutations, while LCNEC may have mutations in TP53, RB1, KRAS, STK11, and other genes. These genetic aspects are essential for understanding their development and creating targeted treatments (25).

HGNEC grow rapidly, are highly invasive, and usually have a poor prognosis. Research into prognostic factors is crucial. A study by Masahiro Morise et al. (26) involving 75 HGNEC patients who had surgery, identified INSM1 as a key prognostic biomarker (27). Another study by the same team linked ALDH1 expression with a poorer prognosis in HGNEC patients. Further research by Sun et al. (28) highlighted significant mutations in genes like TP53, APC, RB1, and CDKN2A in HGNECs. Current research on HGNEC prognosis is limited, emphasizing the need for deeper exploration of the key genetic targets and mechanisms impacting patient outcomes, which is vital for understanding the disease and improving treatments.

In this study, we analyzed gene expression data from HGNEC patients in the GEO database. Our goal was to identify genetic differences between HGNEC tissues with good and poor prognoses. We discovered that SNAP25 is significant in predicting HGNEC outcomes. GO analysis revealed that core genes linked to HGNEC prognosis primarily influence biological functions related to axon development, axonogenesis, neuronal cell bodies, and monoatomic ion channel activity. This suggests their crucial role in nervous system development and their impact on the pathophysiological characteristics and prognosis of HGNEC. Further analysis highlighted that SNAP25 is highly expressed in patients with a poorer prognosis, underscoring its potential importance in understanding and treating HGNEC.

In addition to our primary analysis, we validated our findings using a dataset that included both healthy lung tissues and SCLC samples. This validation revealed that SNAP25 expression is significantly higher in HGNEC patients compared to normal lung tissues. Through ROC curve analysis, we determined that SNAP25 has high diagnostic accuracy. Moreover, our survival prognosis analysis indicates a strong association between increased SNAP25 expression and shorter survival times in HGNEC patients. These consistent findings underscore SNAP25’s vital role as a prognostic biomarker with substantial clinical relevance and potential for application in HGNEC patient care.

SNAP25, a crucial protein in the nervous system, plays a vital role in normal neuronal cell function and synaptic activity. It is key in synaptic vesicle fusion, neurotransmitter release, and modulating synaptic plasticity (29, 30). Abnormal SNAP25 expression or function is linked to neurological disorders (like Alzheimer’s and ADHD) and the development and progression of various neuroendocrine tumors (31, 32). Given its significant expression in neuroendocrine tumors, SNAP25 has been the focus of extensive research. Chen et al. (33) demonstrated SNAP25’s impact on GNEC cell proliferation and chemotherapy resistance, suggesting its potential as a therapeutic target. Di et al. found that reduced SNAP25 (34) expression in prostate cancer correlates with disease progression and immune cell infiltration, emphasizing its value as a prognostic biomarker. Cao et al. ‘s research (11) on lung cancer identified SNAP25-AS1, a related lncRNA, as key in lung cancer metastasis. In hepatocellular carcinoma, Li et al. (12) found an inverse correlation between SNAP25 and the DNA methylation-based stem cell index, indicating its prognostic significance. Hanne-Line Rabben et al.’s study (13) in gastric cancer revealed SNAP25 as a central regulator in tumor metabolism, with its inhibition leading to improved survival in a gastric cancer mouse model. These studies collectively highlight the abnormal expression of SNAP25 across various tumor types, underlining its potential as a prognostic biomarker for multiple cancers.

Our study also explored immune cell infiltration in HGNEC, discovering that SNAP25’s differential expression may affect the prognosis and development of HGNECs. This is achieved by influencing the infiltration of various immune cells, especially M2 macrophages, into the tumor tissue and impacting the tumor microenvironment (TME). We found that SNAP25 may be particularly relevant in SCLC and LCNEC, with its higher expression linked to poorer patient outcomes. However, further research and validation are needed to confirm these findings and to delve deeper into the underlying mechanisms. To validate these observations, we conducted *in vitro* experiments, using techniques like qRT-PCR, Western Blotting, and immunofluorescence. Our results showed a significantly higher expression of SNAP25 in HGNEC cells compared to normal lung epithelial cells and less malignant lung cancer cells. These findings reinforce the link between increased SNAP25 expression and poorer prognosis in HGNEC.

Our study also found that SNAP25 stimulates c-MYC expression through the MEK/ERK signaling pathway. c-MYC, a crucial transcription factor, is involved in numerous biological processes via the MAPK pathway (35, 36). Its persistent activation is linked to cellular reprogramming, increased proliferation, and chemotherapy resistance in various cancers (37). For example, Gao et al.’s research (38) indicated that c-MYC’s high expression promotes breast cancer development by affecting the tumor microenvironment (TME). BRD4 activates c-MYC through transcriptional and epigenetic regulatory mechanisms, thereby increasing the proliferation of gastric cancer cells and inhibiting apoptosis (39). In gastric cancer, BRD4 triggers c-MYC activation, leading to enhanced cell proliferation and reduced apoptosis. c-MYC also regulates key transcription factors like SOX2, POU3F2, and OLIG2, driving the reprogramming of cancer stem cells (CSCs) and fueling cancer progression (40). A recent study by Yuki Oshima et al. (41), examined the prognostic impact of c-MYC expression in HGNECs. Analyzing data from 83 patients, it was found that 33.7% showed positive expression of MYC family proteins (c-MYC, n-MYC, l-MYC), and this was significantly associated with shorter overall survival (OS) and recurrence-free survival (RFS), underscoring the importance of c-MYC in HGNEC prognosis.

Our bioinformatics analysis pinpointed SNAP25 and c-MYC as hub genes linked to prognostic differences in HGNEC, particularly affecting the MAPK signaling pathway. Additionally, in our study, SNAP25 and c-MYC showed notable diagnostic accuracy in ROC analysis for survival prognosis. Based on these results, we hypothesized that SNAP25 might drive HGNEC malignancy by influencing c-MYC through the MAPK pathway. To validate this hypothesis, we conducted a range of *in vitro* experiments. The results from qRT-PCR and Western Blotting showed that, like SNAP25, the expression levels of c-MYC, MEK, and ERK were significantly elevated in HGNEC cells compared to normal lung cells and less malignant lung cancer cells. This evidence supports the idea that SNAP25 could be instrumental in the progression of HGNEC by modulating the c-MYC pathway, thereby playing a crucial role in the disease’s pathogenesis. Furthermore, we constructed a SNAP25 gene knockdown H1299 cell line and verified through CCK8 assay and Transwell experiment that knocking out the SNAP25 gene significantly inhibited the proliferation, migration, and invasion capabilities of HGNEC cells. We also confirmed the RNA and protein expression levels in SNAP25 gene knockdown HGNEC cells through qRT-PCR and Western Blotting experiments. The results showed that in HGNEC cells with SNAP25 gene knockout, the expression of MEK, ERK, and c-MYC was reduced at both RNA and protein levels. This confirms our finding that SNAP25 stimulates c-MYC through the MEK/ERK signaling pathway.

Based on the results, the downregulation of SNAP25 has a pronounced inhibitory effect on the growth of HGNEC xenografts *in vivo*. The substantial reduction in tumor volume observed in the experimental group compared to the control group indicates that SNAP25 plays a critical role in tumor proliferation and survival. These findings suggest that targeting SNAP25 could be a promising therapeutic strategy for HGNEC. The consistent tumor volume measurements further validate the effectiveness of SNAP25 suppression, highlighting its potential as a target for future cancer treatments. Further investigations are warranted to elucidate the underlying mechanisms by which SNAP25 influences tumor growth and to explore the therapeutic applications of SNAP25 inhibitors in clinical settings.

## Conclusion

This study identifies SNAP25 as a key indicator of poor prognosis in HGNEC. SNAP25 influences tumor progression by activating c-MYC through the MEK/ERK pathway. Understanding this SNAP25/MEK/ERK/c-MYC axis broadens our knowledge of HGNEC pathogenesis and opens avenues for targeted therapeutic approaches.

## Data Availability

The datasets presented in this study can be found in online repositories. The names of the repository/repositories and accession number(s) can be found in the article/**Supplementary Material**.

## Supplementary material

Supplementary Table S1 Genes differentially expressed between the Group 1 and Group 2.

Supplementary Table S2 The gene of module red within the GSE9074 dataset.

## References

[1] Kanda Y. Investigation of the freely available easy-to-use software ‘EZR’ for medical statistics. Bone Marrow Transplant. (2013) 48:452–8. doi: 10.1038/bmt.2012.244 PMC3590441 23208313

[2] Travis WD Brambilla E Nicholson AG Yatabe Y Austin JHM Beasley MB. The 2015 world health organization classification of lung tumors: impact of genetic, clinical and radiologic advances since the 2004 classification. J Thorac Oncol. (2015) 10:1243–60. doi: 10.1097/JTO.0000000000000630 26291008

[3] Asamura H Kameya T Fau - Matsuno Y Matsuno Y Fau - Noguchi M Noguchi M Fau - Tada H Tada H Fau - Ishikawa Y Ishikawa Y Fau - Yokose T. Neuroendocrine neoplasms of the lung: a prognostic spectrum. J Clin Oncol. (2006) 24:70–6. doi: 10.1200/JCO.2005.04.1202 16382115

[4] Varlotto JM Medford-Davis LN Fau-Recht A Flickinger JC Zander DS DeCamp MM. Should large cell neuroendocrine lung carcinoma be classified and treated as a small cell lung cancer or with other large cell carcinomas? J Thorac Oncol. (2011) 6:1050–8. doi: 10.1097/JTO.0b013e318217b6f8 21566535

[5] Veronesi G Morandi U Fau - Alloisio M Alloisio M Fau - Terzi A Terzi A Fau - Cardillo G Cardillo G Fau - Filosso P Filosso P Fau - Rea F. Large cell neuroendocrine carcinoma of the lung: a retrospective analysis of 144 surgical cases. Lung Cancer. (2006) 53:111–5. doi: 10.1016/j.lungcan.2006.03.007 16697073

[6] Baker RW Hughson FM. Chaperoning SNARE assembly and disassembly. Nat Rev Mol Cell Biol. (2016) 17:465–79. doi: 10.1038/nrm.2016.65 PMC5471617 27301672

[7] Wang T Li L Hong W. SNARE proteins in membrane trafficking. Traffic. (2017) 18:767–75. doi: 10.1111/tra.2017.18.issue-12 28857378

[8] Yoon TY Munson M. SNARE complex assembly and disassembly. Curr Biol. (2018) 28:R397–401. doi: 10.1016/j.cub.2018.01.005 29689222

[9] Manca P Mameli O Caria MA Torrejón-Escribano B Blasi J. Distribution of SNAP25, VAMP1 and VAMP2 in mature and developing deep cerebellar nuclei after estrogen administration. Neuroscience. (2014) 266:102–15. doi: 10.1016/j.neuroscience.2014.02.008 24534378

[10] Mu Y Yan X Li D Zhao D Wang L Wang X. NUPR1 maintains autolysosomal efflux by activating SNAP25 transcription in cancer cells. Autophagy. (2018) 14:654–70. doi: 10.1080/15548627.2017.1338556 PMC5959327 29130426

[11] Cao Q Dong Z Liu S An G Yan B Lei LA-O. Construction of a metastasis-associated ceRNA network reveals a prognostic signature in lung cancer. Cancer Cell Int. (2020) 20:208. doi: 10.21203/rs.3.rs-20435/v1 32518519 PMC7271455

[12] Li J Zhang C Yuan X Ren Z Yu Z. Correlations between stemness indices for hepatocellular carcinoma, clinical characteristics, and prognosis. Am J Transl Res. (2020) 12:5496–510. PMC7540154 33042433

[13] Rabben HL Andersen GT Olsen MK Øverby A Ianevski A Kainov D. Neural signaling modulates metabolism of gastric cancer. iScience. (2021) 24:102091. doi: 10.1016/j.isci.2021.102091 33598644 PMC7869004

[14] Yu X. A.-O. X. Zhong P Han Y Huang Q Wang J Jia C. Key candidate genes associated with BRAF(V600E) in papillary thyroid carcinoma on microarray analysis. J Cell Physiol. (2019) 234:23369–78. doi: 10.1002/jcp.v234.12 31161615

[15] Zou J Duan D Yu C Pan J Xia J Yang Z. Mining the potential prognostic value of synaptosomal-associated protein 25 (SNAP25) in colon cancer based on stromal-immune score. PeerJ. (2020) 8:e10142. doi: 10.7717/peerj.10142 33150073 PMC7583623

[16] Deng K Guo X Fau - Wang H Wang H Fau - Xia J Xia J. The lncRNA-MYC regulatory network in cancer. Tumour Biol. (2014) 35:9497–503. doi: 10.1007/s13277-014-2511-y 25139102

[17] Bi C Zhang X Chen Y Dong Y Shi Y Lei Y. MAGT1 is required for HeLa cell proliferation through regulating p21 expression. Cell Cycle. (2021) 20:2233–47. doi: 10.1080/15384101.2021.1974792 PMC8794507 34499581

[18] Bigar KK Li SS. Non-histone protein methylation as a regulator of cellular signalling and function. Nat Rev Mol Cell Biol. (2015) 16:5–17. doi: 10.1038/nrm3915 25491103

[19] Zhang ML Zhao TT Du WW Yang ZF Peng W Cui ZJ. C-MYC-induced upregulation of LINC01503 promotes progression of non-small cell lung cancer. Eur Rev Med Pharmacol Sci. (2020) 24:11120–7. doi: 10.26355/eurrev_202011_23599 33215429

[20] Yu G Wang LG Han Y He QY. clusterProfiler: an R package for comparing biological themes among gene. OMICS. (2012) 16:284–7. doi: 10.1089/omi.2011.0118 PMC3339379 22455463

[21] Langfelder P Horvath S. WGCNA: an R package for weighted correlation network analysis. BMC Bioinf. (2008) 29:559. doi: 10.1186/1471-2105-9-559 PMC2631488 19114008

[22] Shimada Y Niho S Ishii G Hishida T Yoshida J M Nishimura M. Clinical features of unresectable high-grade lung neuroendocrine carcinoma diagnosed using biopsy specimens. Lung Cancer. (2012) 75:368–73. doi: 10.1016/j.lungcan.2011.08.012 21920624

[23] He Y Zhao L Tang X Jiang Q Zhao X Cao Y. Prognostic implications of synaptophysin, CD56, thyroid transcription factor-1, and Ki-67 in pulmonary high-grade neuroendocrine carcinomas. Ann Diagn Pathol. (2023) 68:152239. doi: 10.1016/j.anndiagpath.2023.152239 38006863

[24] Malczewska A Kidd M Matar S Kos-Kudła B Bodei L Oberg K. An assessment of circulating chromogranin A as a biomarker of bronchopulmonary neuroendocrine neoplasia: A systematic review and meta-analysis. Neuroendocrinology. (2020) 110:198–216. doi: 10.1159/000500525 31266019

[25] Fernandez-Cuesta L Sexton-Oates A Bayat L Foll M Lau SCM Leal T. Spotlight on small-cell lung cancer and other lung neuroendocrine neoplasms. Am Soc Clin Oncol Educ Book. (2023) 43:e390794. doi: 10.1200/EDBK_390794 37229617

[26] Minami K. Insulinoma-associated protein 1 is a prognostic biomarker in pulmonary high-grade. J Surg Oncol. (2020) 122:243–53. doi: 10.1002/jso.v122.2 32346887

[27] Morise M Hishida T Takahashi A Yoshida J Ohe Y Nagai K. Clinicopathological significance of cancer stem-like cell markers in high-grade neuroendocrine carcinoma of the lung. J Cancer Res Clin Oncol. (2015) 141:2121–30. doi: 10.1007/s00432-015-1985-3 PMC11823834 25963795

[28] Sun TY Zhao L Van Hummelen P Martin B Hornbacker K Lee H. Exploratory genomic analysis of high-grade neuroendocrine neoplasms across. Endocr Relat Cancer. (2022) 29:665–79. doi: 10.1530/ERC-22-0015 PMC10043760 36165930

[29] Hayashi S Hoerder-Suabedissen A Kiyokage E Maclachlan C Toida K Knott G. Maturation of complex synaptic connections of layer 5 cortical axons in the posterior thalamic nucleus requires SNAP25. Cereb Cortex. (2021) 31:2625–38. doi: 10.1093/cercor/bhaa379 PMC8023812 33367517

[30] Wang W Gao W Zhang L Xia Z Zhao B. SNAP25 ameliorates postoperative cognitive dysfunction by facilitating PINK1-dependent mitophagy and impeding caspase-3/GSDME-dependent pyroptosis. Exp Neurol. (2023) 367:114463. doi: 10.1016/j.expneurol.2023.114463 37295545

[31] Najera K Fagan BM Thompson PM. SNAP-25 in major psychiatric disorders: A review. Neuroscience. (2019) 420:79–85. doi: 10.1016/j.neuroscience.2019.02.008 30790667

[32] Noor A Zahid S. A review of the role of synaptosomal-associated protein 25 (SNAP-25) in neurological disorders. Int J Neurosci. (2017) 127:805–11. doi: 10.1080/00207454.2016.1248240 27734716

[33] Chen P. RUNDC3A regulates SNAP25-mediated chemotherapy resistance by binding AKT in gastric neuroendocrine carcinoma (GNEC). Cell Death Discovery. (2022) 8:296. doi: 10.1038/s41420-022-01084-4 35752613 PMC9233710

[34] Di L Gu M Wu Y Liu G Zhang L Li Y. SNAP25 is a potential prognostic biomarker for prostate cancer. Cancer Cell Int. (2022) 22:144. doi: 10.1186/s12935-022-02558-2 35392903 PMC8991690

[35] Saeed H Leibowitz BJ Zhang L Yu J. Targeting Myc-driven stress addiction in colorectal cancer. Drug Resist Update. (2023) 69:100963. doi: 10.1016/j.drup.2023.100963 PMC10330748 37119690

[36] Xu W Gu J Ren Q Shi Y Xia Q Wang J. NFATC1 promotes cell growth and tumorigenesis in ovarian cancer up-regulating c-Myc through ERK1/2/p38 MAPK signal pathway. Tumour Biol. (2016) 37:4493–500. doi: 10.1007/s13277-015-4245-x 26501422

[37] Fatma H Mauraya SK Siddique HR. Epigenetic modifications of c-MYC: Role in cancer cell reprogramming, progression and chemoresistance. Semin Cancer Biol. (2022) 83:166–76. doi: 10.1016/j.semcancer.2020.11.008 33220458

[38] Gao FY Li XT Xu K Wang RT Guan XX. c-MYC mediates the crosstalk between breast cancer cells and tumor microenvironment. Cell Commun Signal. (2023) 21:28. doi: 10.1186/s12964-023-01043-1 36721232 PMC9887805

[39] Ba M. BRD4 promotes gastric cancer progression through the transcriptional and epigenetic regulation of c-MYC. J Cell Biochem. (2018) 119:973–82. doi: 10.1002/jcb.v119.1 28681984

[40] Kozono D Li J Nitta M Sampetrean O Gonda D Kushwaha DS. Dynamic epigenetic regulation of glioblastoma tumorigenicity through LSD1 modulation of MYC expression. Proc Natl Acad Sci U S A. (2015) 112:E4055–64. doi: 10.1073/pnas.1501967112 PMC4522819 26159421

[41] Oshima Y Haruki T Matsui S Makishima K Sakabe T Umekita Y. Clinical significance of MYC family protein expression in surgically resected high-grade neuroendocrine carcinoma of the lung. Thorac Cancer. (2023) 14:758–65. doi: 10.1111/1759-7714.14804 PMC10008680 36694106

