# Supplementary figures and images for: SNAP25-induced MYC upregulation promotes high-grade neuroendocrine lung carcinoma progression
Source: Front Immunol. 2024 Oct 4;15:1411114. doi: 10.3389/fimmu.2024.1411114 (PMC11486671; doi:10.3389/fimmu.2024.1411114)

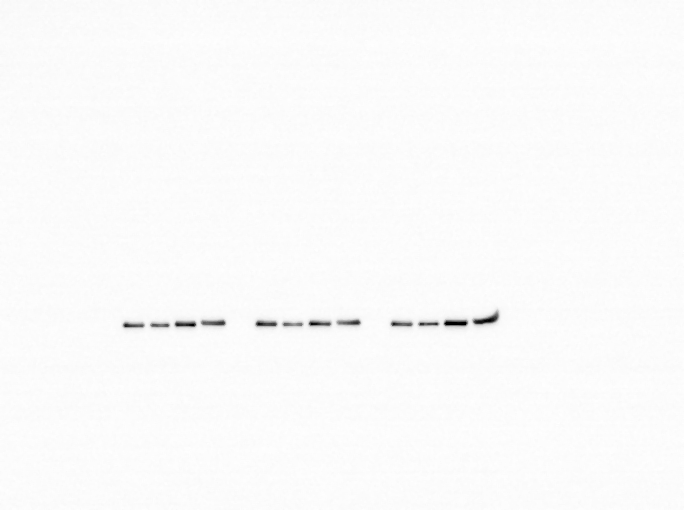

Supplement: Supplementary file 6 [file DataSheet1.zip › Western Bloting of the SNAP25 section that was not knocked/ERK.tif]

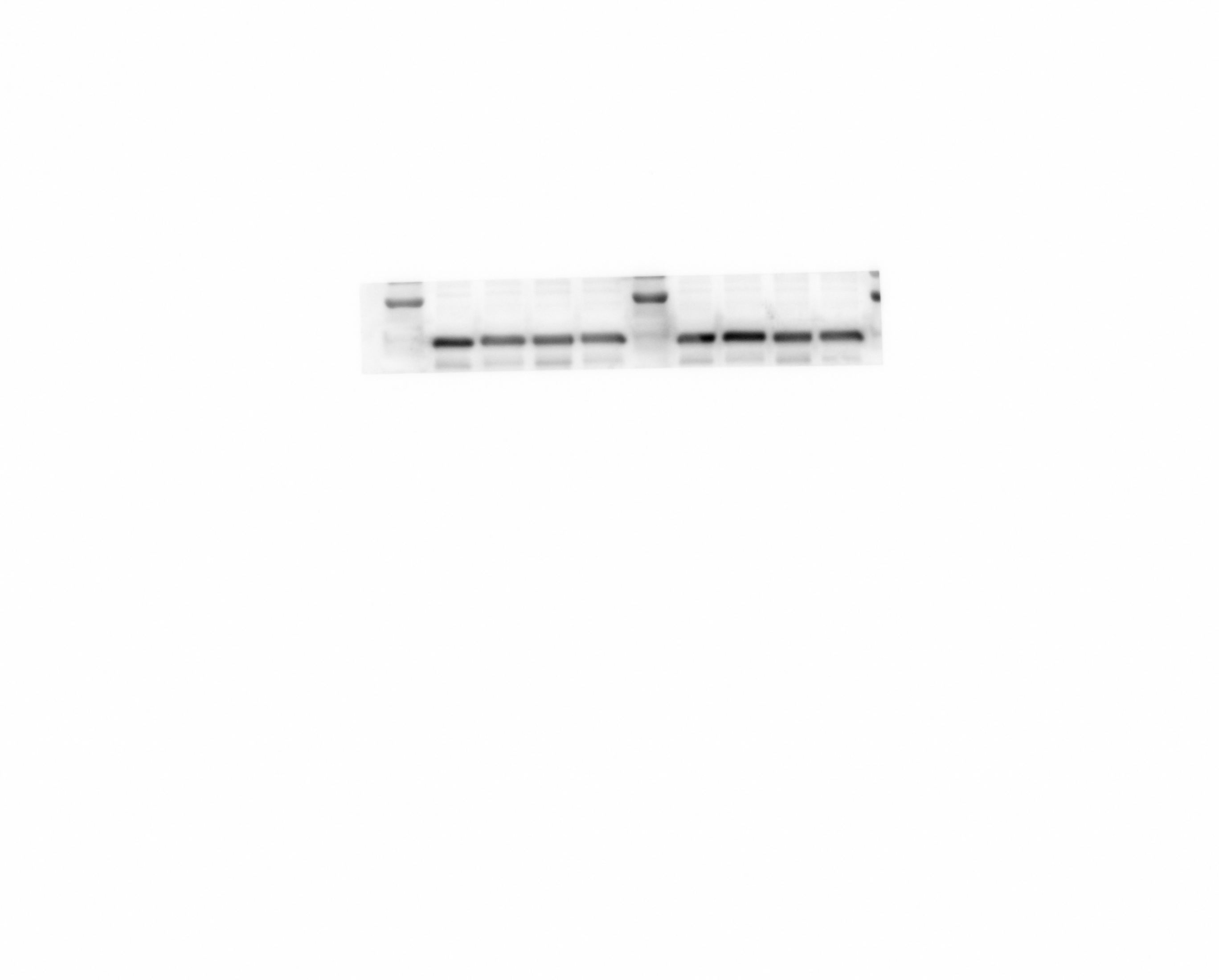

Supplement: Supplementary file 6 [file DataSheet1.zip › Western Bloting of the SNAP25 section that was not knocked/GAPDH.tif]

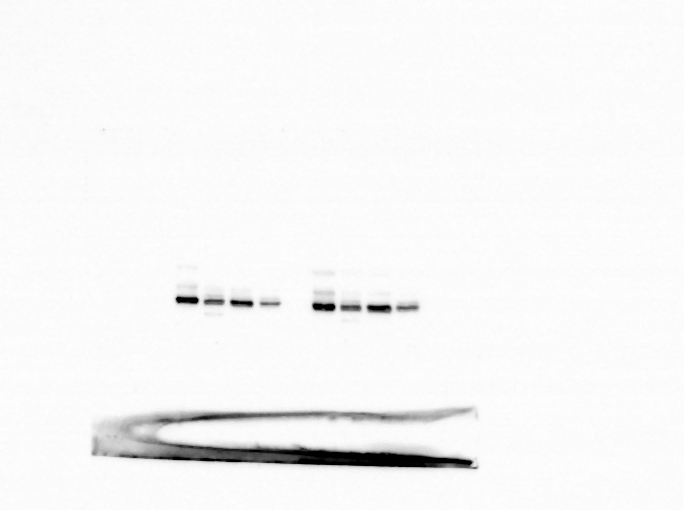

Supplement: Supplementary file 6 [file DataSheet1.zip › Western Bloting of the SNAP25 section that was not knocked/MEK.tif]

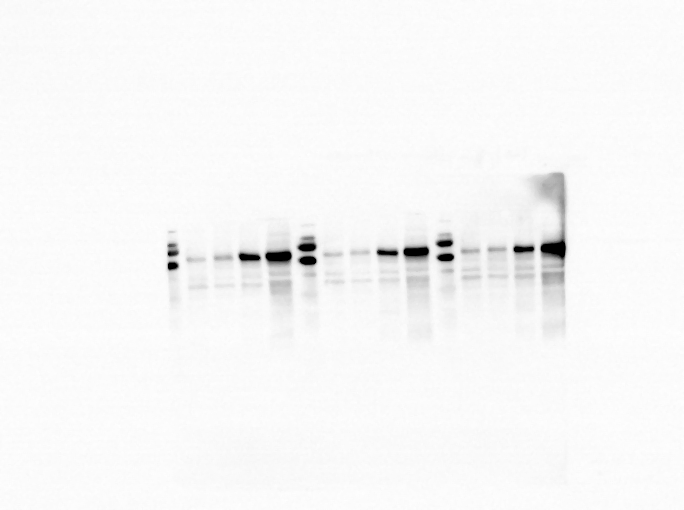

Supplement: Supplementary file 6 [file DataSheet1.zip › Western Bloting of the SNAP25 section that was not knocked/MYC.tif]

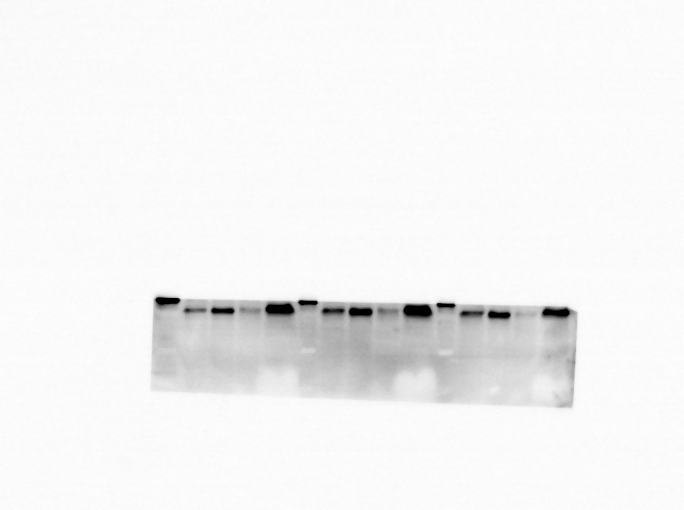

Supplement: Supplementary file 6 [file DataSheet1.zip › Western Bloting of the SNAP25 section that was not knocked/SNAP25.tif]

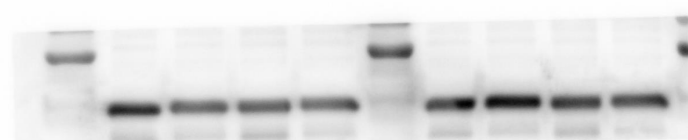

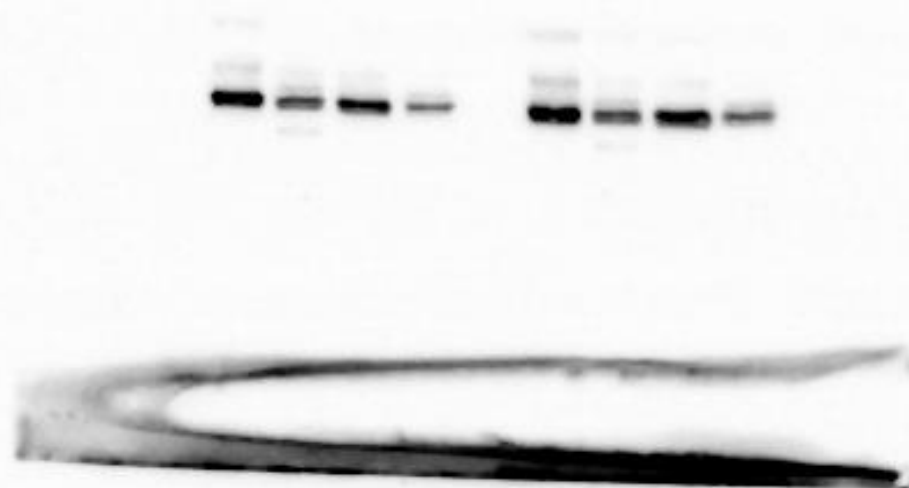

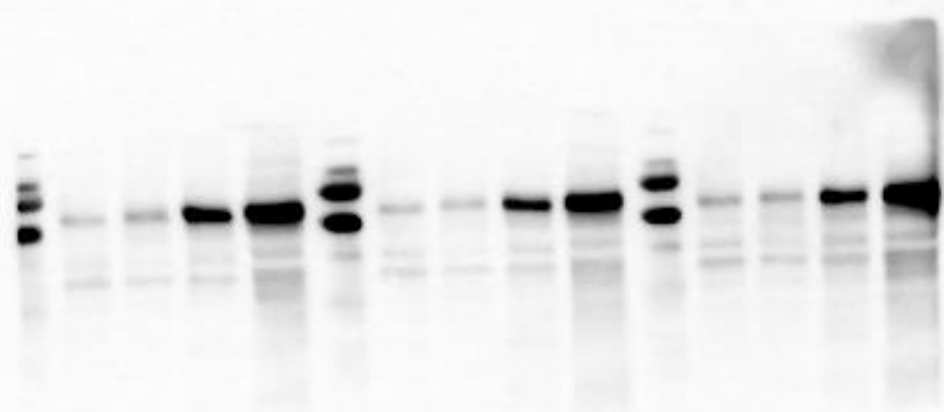

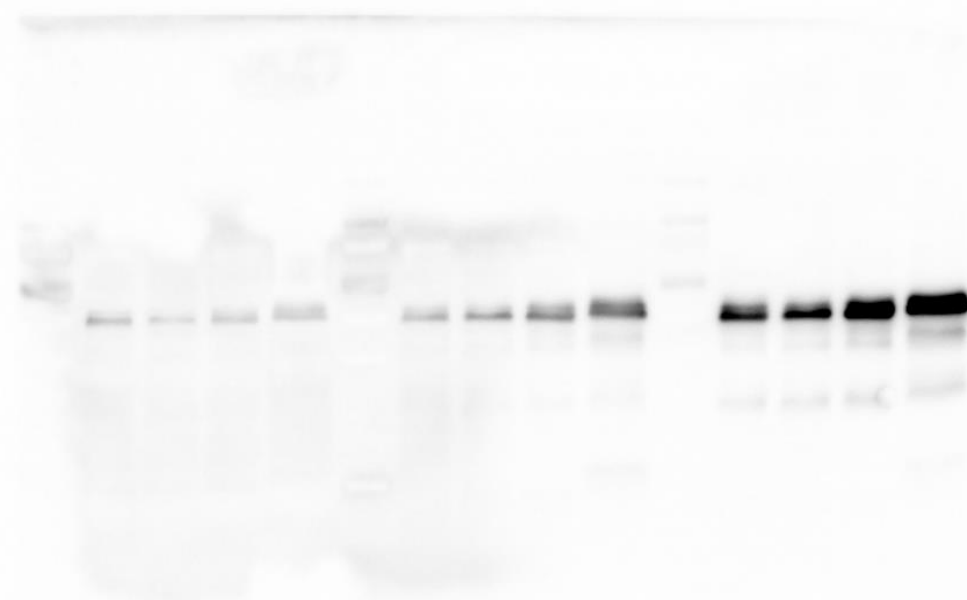

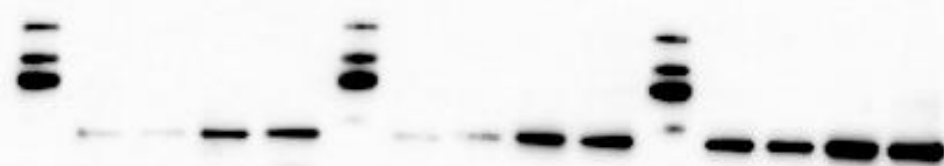

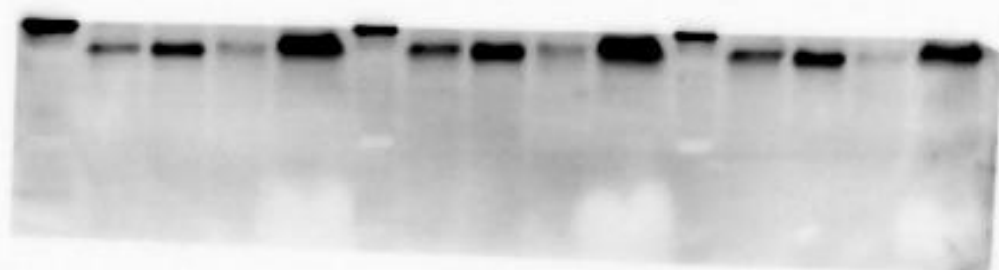

-----

Supplement: Supplementary file 6 [file DataSheet1.zip › Western Bloting of the SNAP25 section that was not knocked/WB1.pdf]

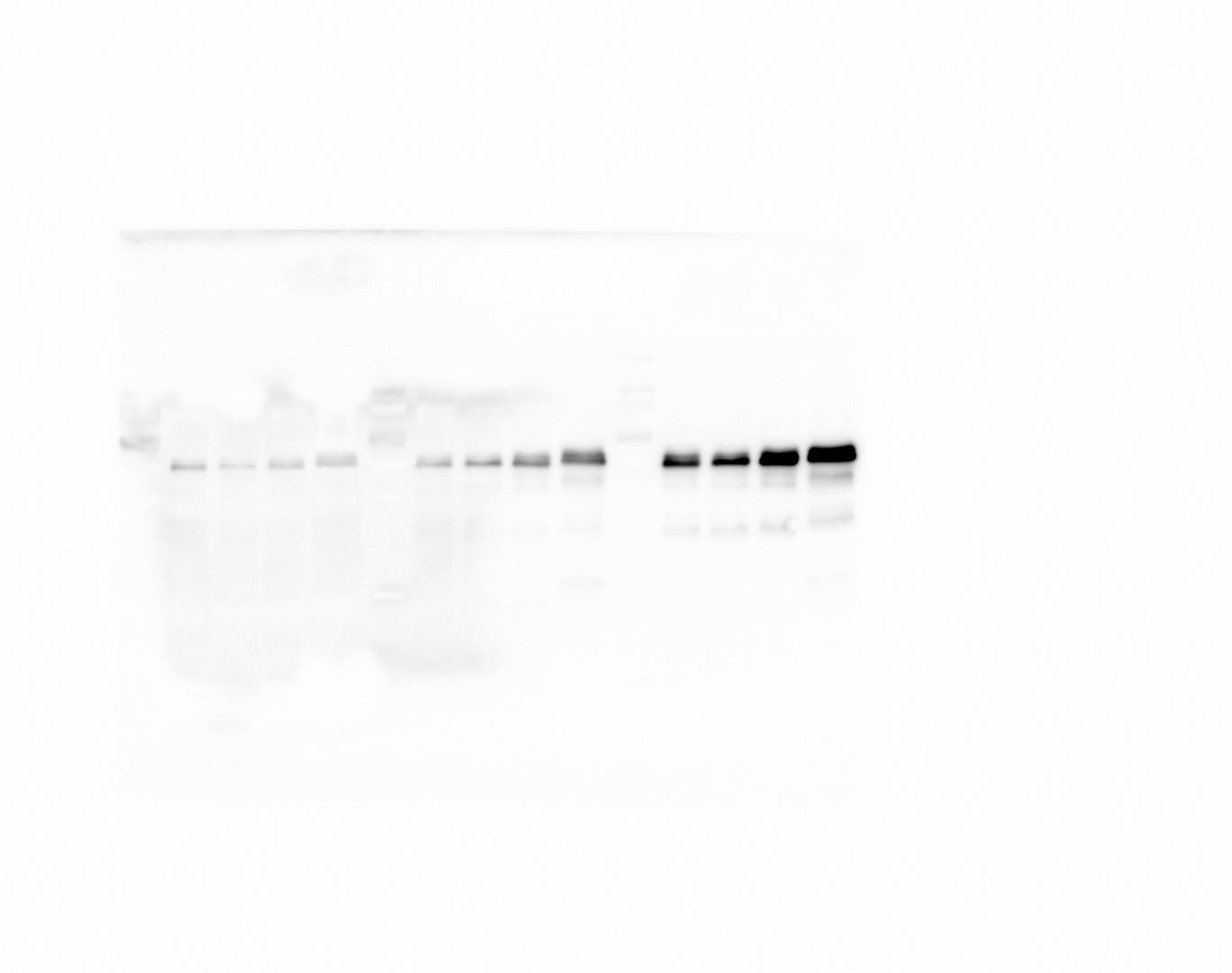

Supplement: Supplementary file 6 [file DataSheet1.zip › Western Bloting of the SNAP25 section that was not knocked/p-ERK.tif]

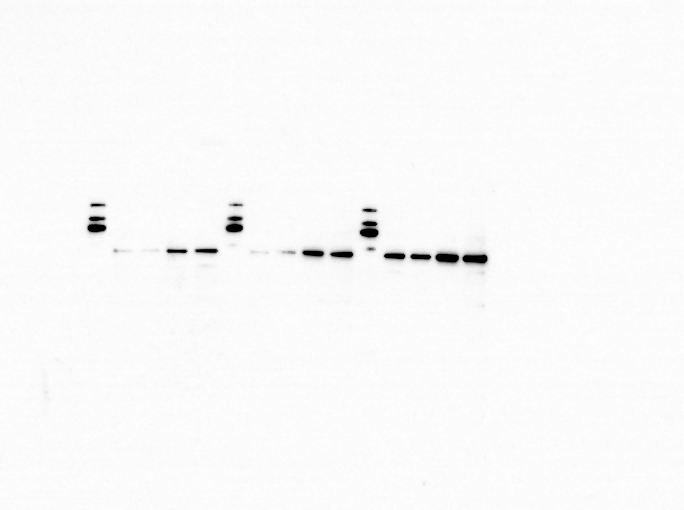

Supplement: Supplementary file 6 [file DataSheet1.zip › Western Bloting of the SNAP25 section that was not knocked/p-MEK.tif]

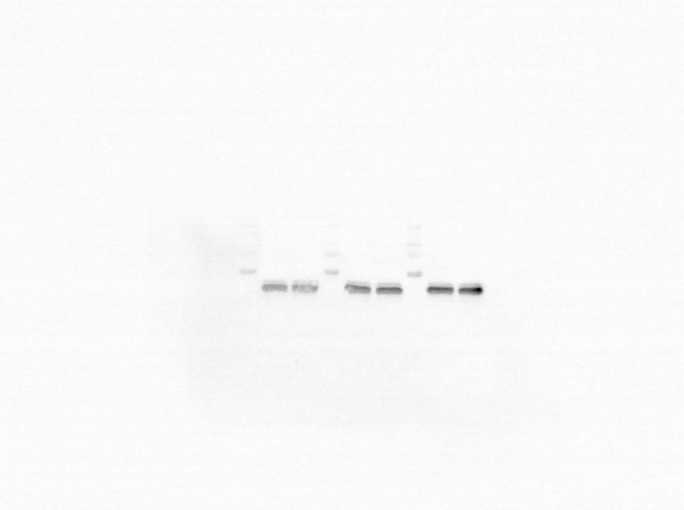

Supplement: Supplementary file 7 [file DataSheet2.zip › Western Bloting of the SNAP25 knockdown section/ERK.tif]

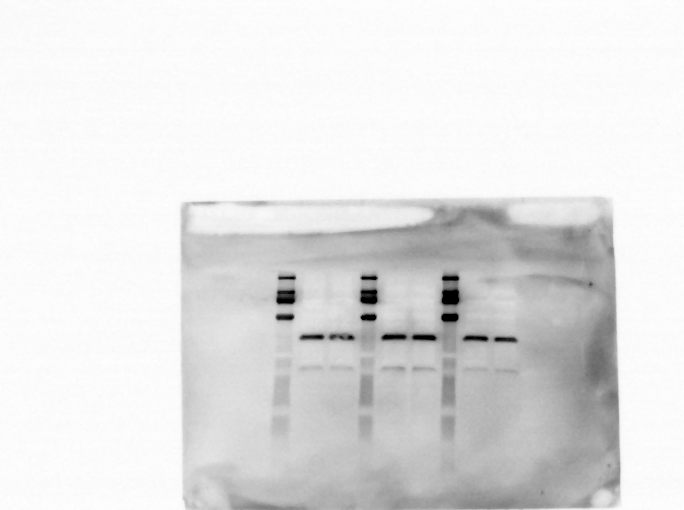

Supplement: Supplementary file 7 [file DataSheet2.zip › Western Bloting of the SNAP25 knockdown section/GAPDH.tif]

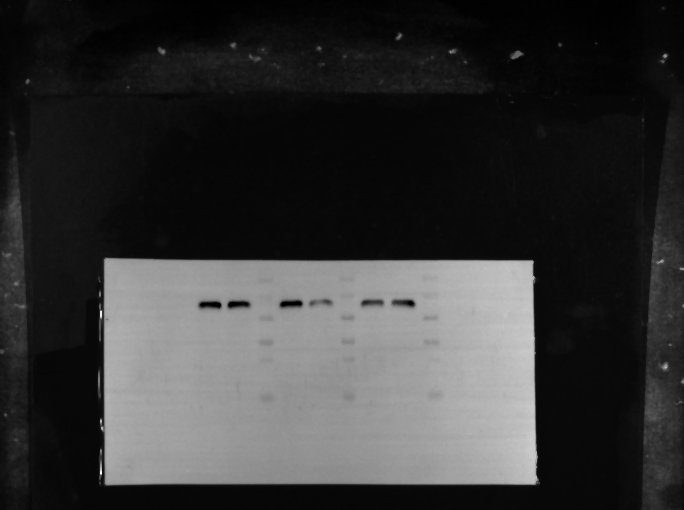

Supplement: Supplementary file 7 [file DataSheet2.zip › Western Bloting of the SNAP25 knockdown section/MEK.tif]

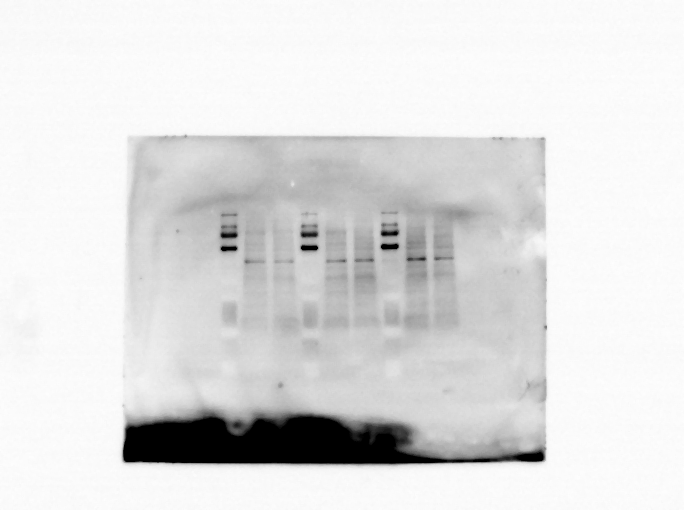

Supplement: Supplementary file 7 [file DataSheet2.zip › Western Bloting of the SNAP25 knockdown section/MYC.tif]

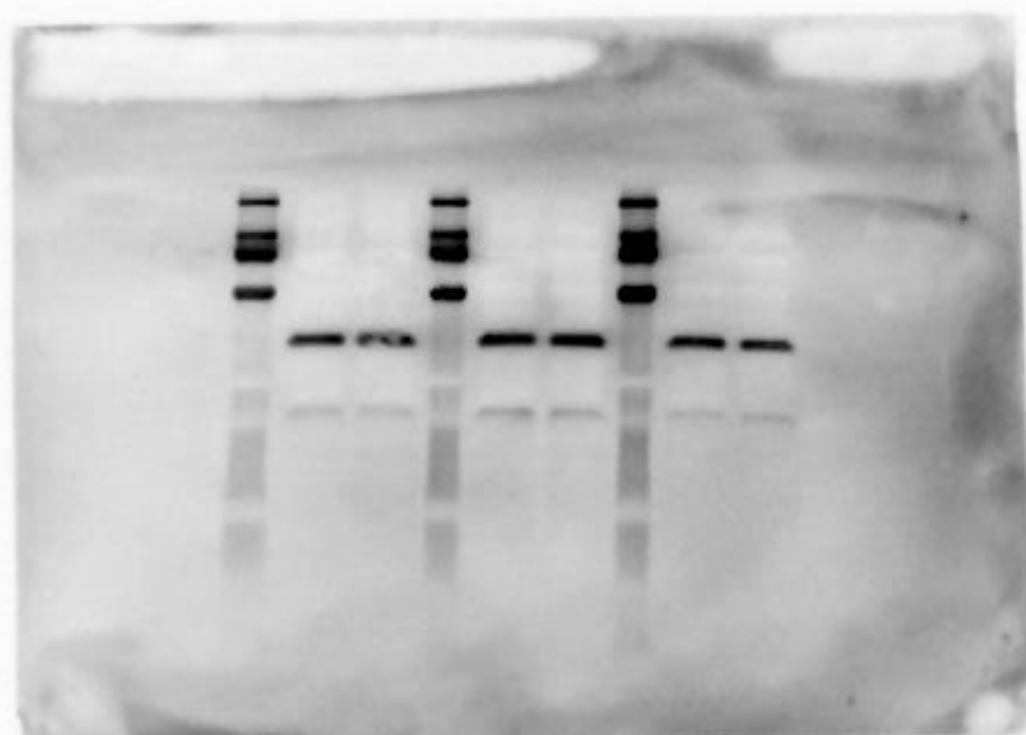

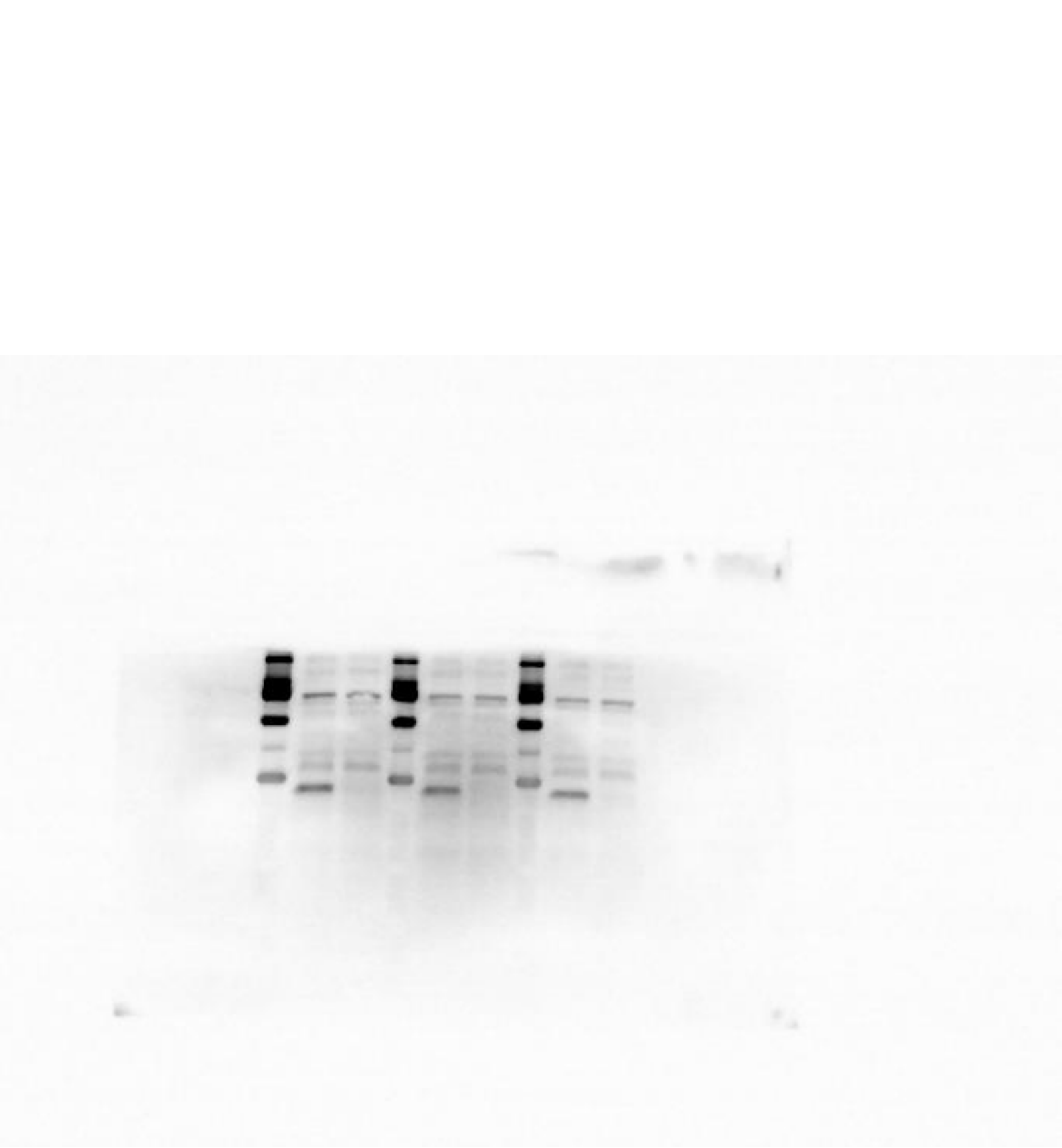

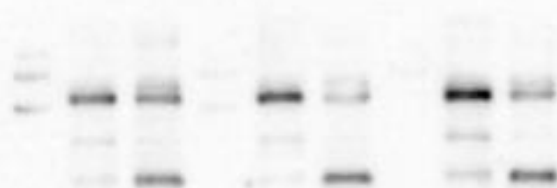

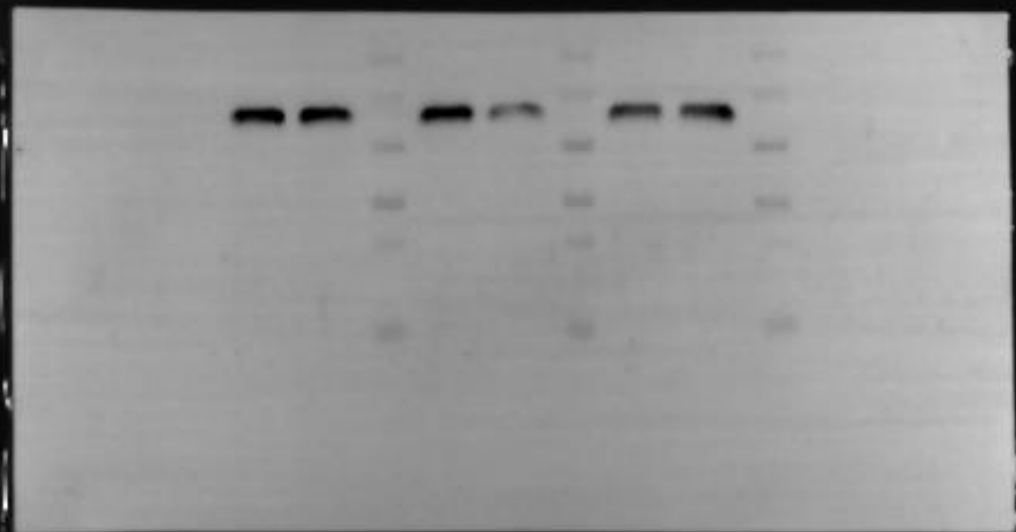

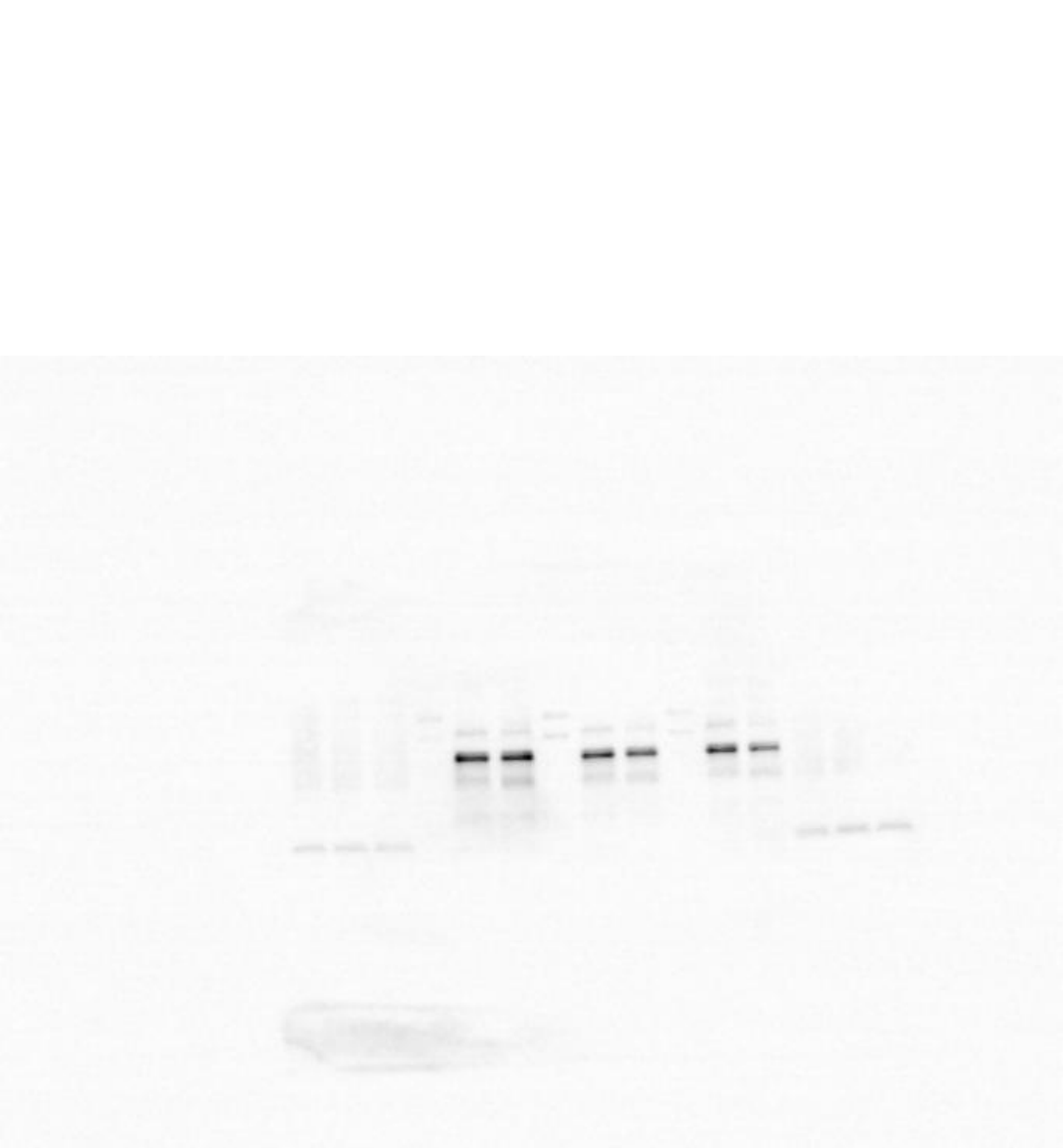



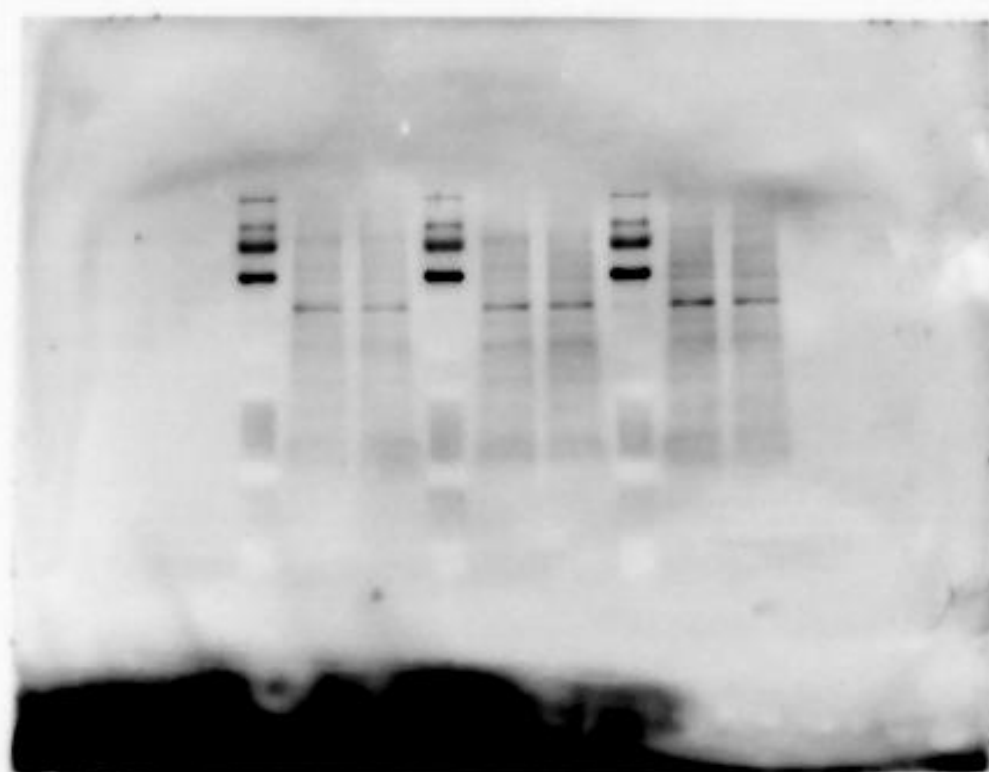

Supplement: Supplementary file 7 [file DataSheet2.zip › Western Bloting of the SNAP25 knockdown section/WB2.pdf]

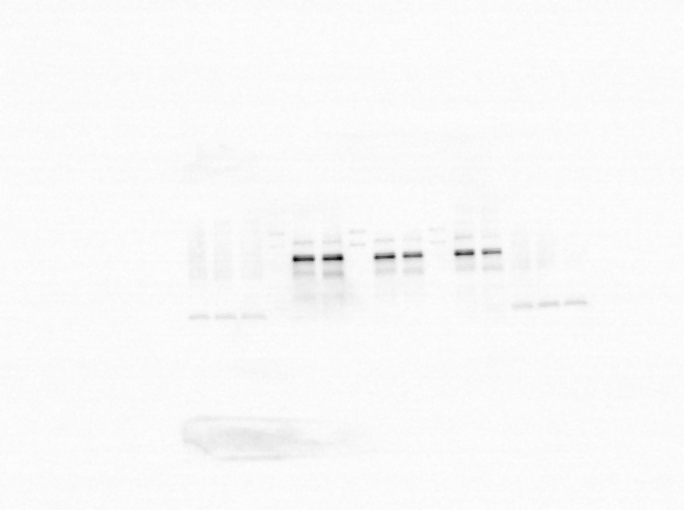

Supplement: Supplementary file 7 [file DataSheet2.zip › Western Bloting of the SNAP25 knockdown section/p-ERK.tif]

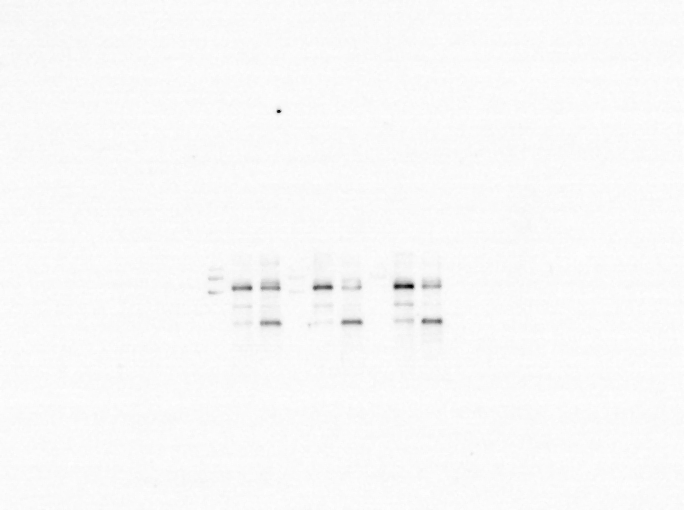

Supplement: Supplementary file 7 [file DataSheet2.zip › Western Bloting of the SNAP25 knockdown section/p-MEK.tif]

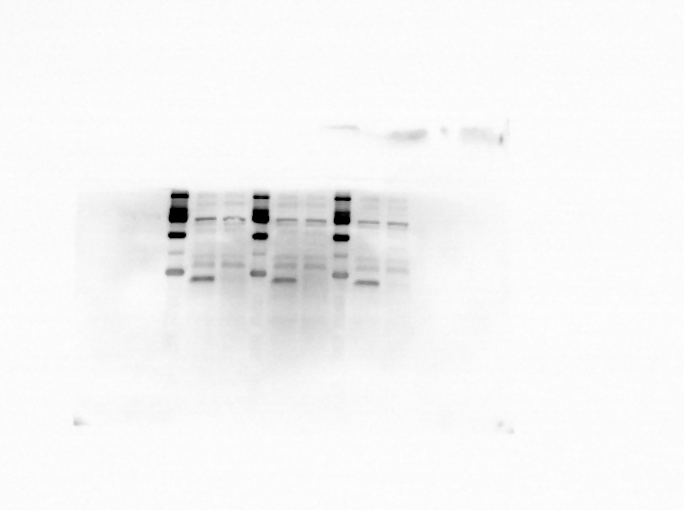

Supplement: Supplementary file 7 [file DataSheet2.zip › Western Bloting of the SNAP25 knockdown section/shSNAP25.tif]
